# A spatial regression model for the disaggregation of areal unit based data to high-resolution grids with application to vaccination coverage mapping

**DOI:** 10.1177/0962280218797362

**Published:** 2018-09-19

**Authors:** CE Utazi, J Thorley, VA Alegana, MJ Ferrari, K Nilsen, S Takahashi, CJE Metcalf, J Lessler, AJ Tatem

**Affiliations:** 1 WorldPop, Department of Geography and Environment, University of Southampton, Southampton, UK; 2 Southampton Statistical Sciences Research Institute, University of Southampton, Southampton, UK; 3 Flowminder Foundation, Stockholm, Sweden; 4 Center for Infectious Disease Dynamics, The Pennsylvania State University, State College, PA, USA; 5 Department of Ecology and Evolutionary Biology, Princeton University, Princeton, NJ, USA; 6 Department of Epidemiology, Johns Hopkins Bloomberg School of Public Health, Baltimore, MD, USA

**Keywords:** Vaccination coverage, spatial misalignment, Bayesian inference, INLA-SPDE, Demographic and Health Surveys

## Abstract

The growing demand for spatially detailed data to advance the Sustainable Development Goals agenda of ‘leaving no one behind’ has resulted in a shift in focus from aggregate national and province-based metrics to small areas and high-resolution grids in the health and development arena. Vaccination coverage is customarily measured through aggregate-level statistics, which mask fine-scale heterogeneities and ‘coldspots’ of low coverage. This paper develops a methodology for high-resolution mapping of vaccination coverage using areal data in settings where point-referenced survey data are inaccessible. The proposed methodology is a binomial spatial regression model with a logit link and a combination of covariate data and random effects modelling two levels of spatial autocorrelation in the linear predictor. The principal aspect of the model is the melding of the misaligned areal data and the prediction grid points using the regression component and each of the conditional autoregressive and the Gaussian spatial process random effects. The Bayesian model is fitted using the INLA-SPDE approach. We demonstrate the predictive ability of the model using simulated data sets. The results obtained indicate a good predictive performance by the model, with correlations of between 0.66 and 0.98 obtained at the grid level between true and predicted values. The methodology is applied to predicting the coverage of measles and diphtheria-tetanus-pertussis vaccinations at 5 × 5 km^2^ in Afghanistan and Pakistan using subnational Demographic and Health Surveys data. The predicted maps are used to highlight vaccination coldspots and assess progress towards coverage targets to facilitate the implementation of more geographically precise interventions. The proposed methodology can be readily applied to wider disaggregation problems in related contexts, including mapping other health and development indicators.

## 1 Introduction

The launch of the Sustainable Development Goals (SDGs) in 2015^1^ with the central focus of ‘leaving no one behind’ has prompted a call for spatially detailed data to improve the evaluation and monitoring of key health and development measures within countries. High-resolution maps of development and health indicators are useful tools for determining the geographical variation and inequities in these indicators to better inform decision making, policy and targeting of interventions. Maps built on geolocated household survey data integrated with geospatial covariates have grown in popularity in recent years^2–5^ due to their advantages over small area estimation,^6–8^ including flexibility for use in monitoring progress towards development goals at more operationally relevant spatial scales and non-reliance on additional data from censuses or other administrative sources. Central to their production are data obtained from national household surveys, such as the Demographic and Health Surveys (DHS),^9^ which are typically conducted every 3–5 years in low- and middle-income countries to provide data on a wide range of relevant indicators. Many DHS surveys (as well as surveys from other programs) are now geolocated, and the increasing availability of the global positioning system (GPS) coordinates of survey clusters have facilitated the integration of cluster-level data with geospatial covariate layers, often in a model-based framework, to map these indicators. Geostatistical models which characterize spatial dependence via parametric covariance functions,^10^ and generalized additive models^11^ utilizing smooth functions of the cluster coordinates to model spatial autocorrelation, are commonly used approaches. However, the feasibility of these approaches rely on the availability of GPS-located cluster centroid data.

In some cases, access to national survey data sets with GPS cluster location data can be limited, due to various reasons including security, confidentiality and political concerns. Therefore, in certain countries, data from these surveys can at best be obtained at an aggregate level, typically at the first administrative level (i.e. provinces). Consequently, high-resolution mapping methods which are designed for point-referenced data cannot be applied. Providing local-scale subnational estimates to better guide health interventions,^11,12^ therefore, requires alternative methods for dealing with the problem of spatial misalignment that exists between the accessible subnational (or areal) data and the grid points at which predictions are required.

Spatial misalignment or change of support problems is well-studied in the statistical literature.^13,14^ Four types of misalignment are often encountered in practice: (i) area-to-area (also known as modifiable areal unit problem),^15,16^ (ii) area-to-point,^17,18^ (iii) point-to-point and (iv) point-to-area. These misalignment problems represent many contexts where data are available at a given spatial scale (or multiple scales), whereas inference or predictions are required at another scale that represents a completely different spatial configuration.^18,19^ Methods for point-to-point and point-to-area misalignment constitute the crux of geostatistical studies.^10^ Many model-based approaches, some of which are tailored to certain applications, have also been developed for dealing with other misalignment problems.^13,14^ These are mostly implemented in a Bayesian framework using Markov chain Monte Carlo (MCMC) methods, although the Integrated Nested Laplace Approximations (INLA) method^20^ is becoming popular recently. Nevertheless, methods for area-to-area and area-to-point misalignment, especially with non-Gaussian outcomes, are less frequently studied and most existing approaches are not available in commonly used software packages.

The primary objective of this paper is to develop a novel approach to the area-to-point disaggregation problem, focusing on high-resolution mapping of childhood vaccination coverage using areal survey data. The proposed approach is a joint model that combines a conditional autoregressive (CAR) model for the observed areal data and a Gaussian process model for the prediction grids, whilst intrinsically adjusting for the misalignment in the covariates included in the model. A key aspect of our hierarchical modelling strategy is the linking of the areal observations and the prediction grids using these latent processes and the regression component. The Bayesian model is fitted using the INLA method. INLA is a deterministic algorithm that utilizes both analytical approximation and numerical integration to perform approximate Bayesian inference for the class of latent Gaussian models, which includes spatial and spatiotemporal models. As a faster and accurate alternative to simulation-based MCMC methods, the INLA approach has gained popularity among researchers partly due to the availability of the R-INLA package for its implementation.^21,22^ To implement the INLA approach for point-referenced data, it is often combined with the stochastic partial differential equation (SPDE) approach proposed by Lindgren et al.^23^

The remainder of this paper is structured as follows. The data sets analyzed – vaccination coverage data and the prediction covariates – are discussed and displayed in Section 2. The proposed model and the accompanying Bayesian inferential procedure using the INLA-SPDE approach are discussed in Section 3. In Section 4, a simulation study is carried out to examine the predictive performance of the model under different scenarios. An application to high-resolution mapping of vaccination coverage in Afghanistan and Pakistan is presented in Section 5. As a further validation exercise, in Section 6, predicted maps produced using the proposed methodology are compared with those obtained via geostatistical approaches that utilize geolocated cluster level data, based on parallel data sets containing both areal and geolocated cluster level information. We conclude with some discussion in Section 7.

## 2 Data

Subnational vaccination coverage data for Afghanistan and Pakistan were obtained from the most recent DHS surveys conducted in 2015 and 2013, respectively, in both countries.^24,25^ For Afghanistan, the subnational areas were the 34 provinces of the country whereas for Pakistan, these were the eight administrative level 1 areas (although the survey excluded Azad Kashmir and Federally Administered Tribal Areas (FATA)). When obtaining aggregate summaries of DHS data, it is required that sampling weights are applied to account for the survey design.^26^ Hence, the subnational data used here were weighted to adjust for the selection probabilities and non-response. Other information related to the surveys including the population sizes of the subnational areas can be found in the relevant DHS reports.^24,25^

For each area in both countries, data on measles and diphtheria-tetanus-pertussis (DTP) vaccinations were extracted and matched to the corresponding boundaries obtained from DHS spatial data repository.^27^ The data for measles vaccination coverage, by definition,^24,25^ refer to coverage with at least the first dose of measles containing vaccine (MCV1), which is usually administered from age 9 months. For DTP, the coverage of each of the three doses: DTP1, DTP2 and DTP3, recommended at 6, 10 and 14 weeks, respectively, was obtained separately. For each vaccination type, the data comprised of the numbers of children aged 12–23 months (a standard age group for assessing vaccination coverage, see literature^24,25^) surveyed and the numbers that were vaccinated at any time prior to the survey. Whether or not a child was vaccinated was determined during the surveys either from the child's vaccination card or through parental recall. We note that there is a potential for information bias associated with determining vaccination status through parental recall in the absence of vaccination cards. However, analysis of cards only data is constrained by sample size issues due to high proportions of children without vaccination cards in both countries (≈67% in Pakistan).^24,25^

Overall, the weighted data comprised of 5704 children in Afghanistan, of which 3443 (60.4%), 4166 (73.0%), 3875 (67.9%) and 3293 (57.7%) had received measles and DTP 1, 2, 3 vaccinations, respectively. For Pakistan, out of 2074 children, 1274 (61.4%), 1633 (78.7%), 1508 (72.7%) and 1352 (65.2%) had received the respective vaccinations. The province of Zabul in Afghanistan was excluded from all the summary tables of indicators produced following the 2015 DHS survey^24^ due to poor accessibility during the survey. This area and the excluded provinces in Pakistan were treated as missing data in the analysis.

Geospatial socioeconomic, demographic, environmental and physical factors play an important role in determining the spatial patterns and geographic inequities in vaccination coverage.^12^ As such, these have been used in previous research to map vaccination coverage at fine spatial scales. To inform our disaggregation model, we selected two covariates from a suite of geospatial covariate layers available for both countries through the WorldPop project (www.worldpop.org.uk). These were: travel time to major cities of at least 50,000 people^28^ and population density.^29^ This number of covariates was chosen deliberately to guard against the possibility of overfitting. The selected covariate layers were each preprocessed and resampled to match the administrative boundary shapefiles of both countries and the 5 km prediction grids using ArcGIS v10.4.

**Figure 1.**
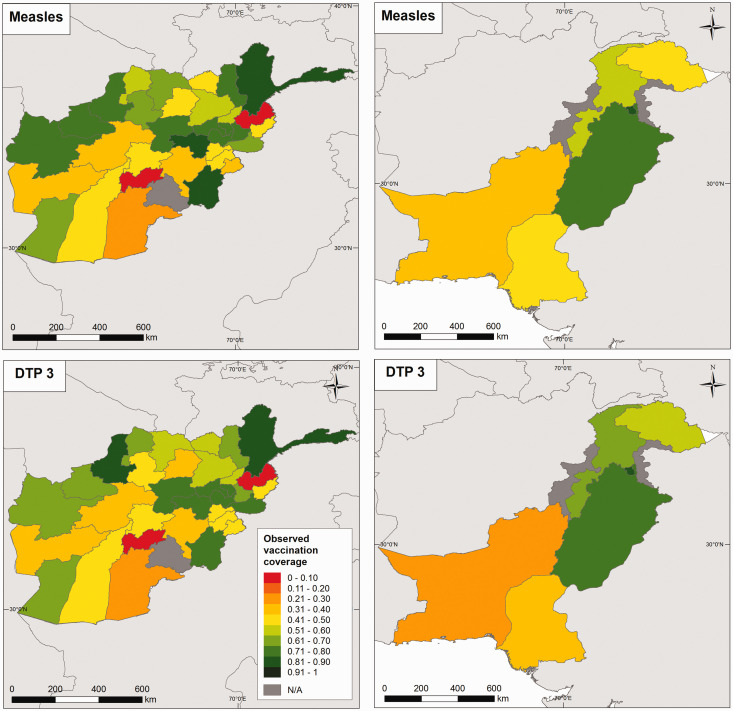
Maps of observed measles and DTP3 vaccination coverage for Afghanistan in 2015 (left panel) and Pakistan in 2013 (right panel) at administrative level 1.

In Figure 1, we show the observed vaccination coverage data for measles and DTP3 for both countries. Similar maps for DTP1 and DTP2 and the maps of the covariate layers are displayed in online Supplemental Figures S1 and S2, respectively.

## 3 Methods

### 3.1 The disaggregation model

The disaggregation model used in this work is formalized as follows. Let $A\in {\mathbb{R}}^{2}$ denote the study regions of Afghanistan and Pakistan, each of which is partitioned into $n_{A}$ areal units (or subnational areas) $A_{1},\ldots ,A_{n_{A}}$ . The aim is to predict the quantity of interest $p_{i}$ , the probability of being vaccinated at location *i*, over a set of $n_{p}$ grid points $s_{1},\ldots ,s_{n_{p}}$ . Let $Y_{i}$ denote the number of children vaccinated within area $A_{i}$ or at grid point $s_{i}$ and $N_{i}$ , the corresponding number of children surveyed. We note that both $Y_{i}$ and $N_{i}$ are unobserved at the grid point level. The proposed model is given by

(1) Yi∼Binomial(Ni,pi),i=1,…,nA,nA+1,…,nA+nplogit(pi)=x∼i'β+|Ai|-1∫Aiη(s)ds+φi,i=1,…,nAlogit(pi)=xi'β+η(si)+φAi,i=nA+1,…,nA+np

In equation (1), $x_{i}$ and ${\overset{∼}{x}}_{i}$ are *k* × 1 vectors of covariate values for the *i*th area and grid point, respectively, both of which include an intercept term, while ***β*** are the corresponding regression coefficients. To deal with the misalignment between the observation areas and the deterministic gridded prediction covariates, $x_{i}(i=n_{A}+1,\ldots ,n_{A}+n_{p})$ , the covariate values for each area were obtained as *block averages* of the corresponding grid point values within the area, so that ${\overset{∼}{x}}_{i}=|A_{i}{|}^{-1}\int_{A_{i}}x(s)\text{d}s$ for $i=1,\ldots ,n_{A}$ , where, as in equation (1), $|A_{i}|=\int_{A_{i}}\text{d}s$ is the size of the *i*th area. This provides the rationale for the specification of a joint set of regression coefficients in the model. Other terms in the model are explained as follows. $\eta =(\eta (s_{n_{A}+1}),\ldots ,\eta (s_{n_{A}+n_{p}}))'$ are spatial random effects characterizing spatial autocorrelation at the grid point level in the model. These are assumed to have arisen from a zero-mean stationary Gaussian process, that is η∼N(0,Σ) , where Σ is a spatially structured, positive definite covariance matrix. Specifically, Σ is assumed to follow the Matérn^30^ class of covariance functions such that for generic grid points $s_{i}$ and $s_{j}\in {\mathbb{R}}^{2}$ , we have that

(2) $$\Sigma_{\mathrm{ij}}=\text{Cov}(\eta (s_{i}),\eta (s_{j}))=\frac{\sigma_{\eta }^{2}}{2^{\nu -1}\Gamma (\nu )}(\kappa \|s_{i}-s_{j}\|{)}^{\nu }K_{\nu }(\kappa \|s_{i}-s_{j}\|)$$

where ‖.‖ denotes the Euclidean distance, $\sigma_{\eta }^{2}$ is the marginal variance of the process, κ is a scaling parameter related to the range $r(r=\frac{\sqrt{8\nu }}{\kappa })$ – the distance at which spatial correlation is approximately 0.13, and $K_{\nu }$ is the modified Bessel function of the second kind and order ν>0 .^31^ It is often the practice to fix the smoothness parameter ν due to identifiability issues. Here, we set ν=1 , see Lindgren et al.^23^

The second set of spatial random effects $\varphi =(\varphi_{1},\ldots ,\varphi_{n_{A}})'$ capture spatial autocorrelation in the observed areal data, and are assigned a conditional autoregressive (CAR) prior, a special case of Gaussian Markov Random Field (GMRF) models popularly used in disease mapping studies.^32^ Here, we assume the CAR model proposed by Leroux et al.^33^ which was used in a similar setting in Napier et al.^34^ A recent study^35^ found that this CAR model outperformed other choices often used in disease mapping studies. The model is given by $\varphi ∼N(0,\sigma_{\varphi }^{2}Q^{-1}(W))$ , where $Q(.{)}_{n_{A}\times n_{A}}$ is a precision matrix and $\sigma_{\varphi }^{2}$ is a variance parameter. More explicitly, $Q(W)=\rho (\text{diag}(W1)-W)+(1-\rho )I_{n_{A}}$ , where ρ is a spatial autocorrelation parameter, **1** is an $n_{A}$ vector of 1's, $I_{n_{A}}$ is the identity matrix and W is a binary matrix characterizing the neighbourhood structure of the areas. That is $W_{\mathrm{ij}}=1$ if areas $A_{i}$ and $A_{j}$ share a common border and zero otherwise. The additional modelling in the second and third levels of equation (1) using $\varphi_{A_{i}}$ , which denotes the value of φ corresponding to the area to which the *i*th grid cell belongs, and the areal averages of ***η*** demonstrate clearly the role of these random effects in melding the two spatial scales in the model.

### 3.2 Bayesian inference using the INLA-SPDE approach

We propose to fit the model in equation (1) using the INLA-SPDE approach.^20,23^ Let $\theta =(\beta ,\sigma_{\eta }^{2},\kappa ,\sigma_{\varphi }^{2},\rho )'$ denote the vector of parameters of the model. The joint posterior distribution (with augmented data likelihood) is proportional to:

$$\overset{n_{A}+n_{p}}{\underset{i=1}{\Pi }}\{\text{Binomial}(Y_{i};N_{i},p_{i})\}\times N(\eta ;0,\Sigma )\times N(\varphi ;0,\sigma_{\varphi }^{2}Q^{-1}(W))\times p(\theta )$$

where *p*(***θ***) is the joint prior distribution of ***θ***. The INLA approach produces a numerical approximation of the marginal posterior distributions of each element of ***θ***, using the Laplace approximation method. Following internal parameterizations in R-INLA, we placed the following noninformative and, in some cases, weakly informative priors on the parameters: $\beta ∼N(0,10^{5}I)$ , $\text{log}(1/\sigma_{\varphi }^{2})∼\text{logGamma}(1,0.01)$ , log(ρ/(1-ρ))∼N(0,0.45) and $\text{log}(\kappa )∼N(\text{log}(\sqrt{8}/m),1)$ , where *m* is the median distance between the prediction grids. A default non-informative prior was assumed for $\sigma_{\eta }^{2}$ (see Blangiardo and Cameletti^21^ for details).

The SPDE approach is particularly required for the estimation of the latent Gaussian field, ***η***. The approach entails the representation of the field using a discretely indexed GMRF, constructed via a linear fractional SPDE^21^ which has the Gaussian field ***η*** with the Matérn covariance function as its exact solution. This solution is approximated using the finite element method through a basis function representation defined on a triangulation of the study region, A , given by

(3) $$\eta (s)=\sum_{g=1}^{G}\psi_{g}(s){\overset{∼}{\eta }}_{g}$$

where *G* is the number of vertices in the triangulation, $\{\psi_{g}\}$ are basis functions that are piecewise linear in each triangle (i.e. $\psi_{g}$ is 1 at vertex g and 0 at all other vertices) and $\left\{{\overset{∼}{\eta }}_{g}\right\}$ are zero mean Gaussian-distributed weights.^23^ Thus, for the *i*th grid location, we have that $\eta (s_{i})=\sum_{g=1}^{G}\psi_{g}(s_{i}){\overset{∼}{\eta }}_{g}=\sum_{g=1}^{G}A_{\mathrm{ig}}{\overset{∼}{\eta }}_{g}$ , where A is an $n_{p}\times G$ sparse matrix that maps the GMRF $\left\{{\overset{∼}{\eta }}_{g}\right\}$ ^23^ from the *G* triangulation nodes to the $n_{p}$ grid points. With the choice of basis functions in equation (3), the Gaussian weights determine the value of η(s) for grid points coinciding with the vertices (since $A_{\mathrm{ig}}=1$ if ***s***_*i*_ is at the vertex), and the values of the points in the interior of the triangles are determined by linear interpolation. Although the ***A*** matrix, also known as the projection matrix, is mostly used for handling point-referenced data, the R-INLA function ‘*inla.spde.make.A*’ contains additional arguments that allow the evaluation of the term $|A_{i}{|}^{-1}\int_{A_{i}}\eta (s)ds$ in equation (1) for each area. This consists in the approximation of the integral of the process for each area by averaging over all the vertices weights within the area. That is, $|A_{i}{|}^{-1}\int_{A_{i}}\eta (s)ds\approx \sum_{g=1}^{G}A_{\mathrm{ig}}{\overset{∼}{\eta }}_{g}$ , where $A_{\mathrm{ig}}=1/V_{i}$ if vertex *g* is in area $A_{i}$ (and zero otherwise), $V_{i}$ is the number of vertices in the area and ***A*** is now an $n_{A}\times G$ matrix. Hence, it is necessary to define a fine triangulation of the domain A in order to minimize the error due to this approximation; see Moraga et al.^18^ and online Supplemental materials for details. The model set-up in equation (1) implies that the INLA algorithm generates predictions for the target grid locations during model-fitting. Any missing areal data are also estimated similarly.

The R code for the analysis is provided in the online Supplemental materials.

## 4 Simulation study

**Figure 2.**
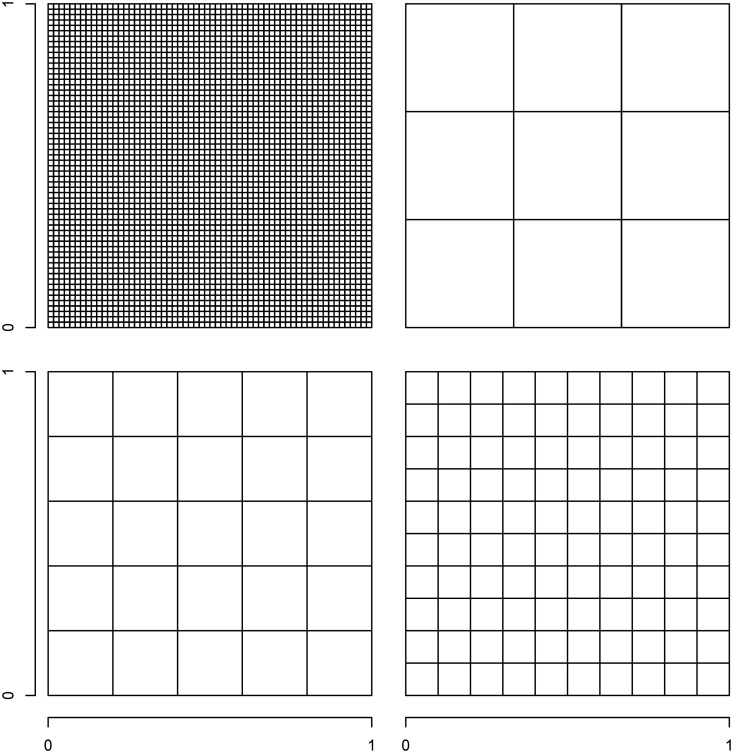
Plots of the grid ( $n_{p}$ = 3600) and areal configurations ( $n_{A}$ = 9,25,100) used in the simulation study. These were generated on the unit (i.e. [0,1] × [0,1]) square.

The purpose of this simulation study is to demonstrate the predictive ability of the proposed model in scenarios depicting the intended applications. Data were generated using the unit (i.e. [0,1]×[0,1] ) square as the study region. The prediction grid was generated as a 60×60 (i.e. $n_{p}=3600$ ) raster over the square while the observation areas were obtained by partitioning the square into $n_{A}=9,25$ and 100 square areas. These areal and grid configurations are plotted in Figure 2.

The true values of the parameters of model (1) used in generating the data are described as follows. For the spatial process ***η***, we set $\sigma_{\eta }^{2}=1.0$ and varied its range relative to the size of the study region to investigate the effect of varying degrees (low to high) of spatial dependence on the predictions. The chosen values were *r* = 0.3, 0.5, 0.7, corresponding to *κ* = 9.4, 5.7, 4.0. The spatial random effect, φ, was generated from the Normal distribution given in Section 3.1 with the parameter values: ρ=0.6 and $\sigma_{\varphi }^{2}=1$ . For the regression coefficients, we have β=(0.2,0.4,-0.5,0.2,-0.2) , with the covariate vector comprising an intercept term and four variables simulated from N(0,1) , Gamma (1,1), Poisson (5) and *t*(2), to illustrate different types of covariate factors that could be encountered in practice. (We note that smaller numbers of covariates such as k=2 yielded similar results as these.) Sample sizes for the observation areas, that is $N_{1},\ldots ,N_{n_{A}}$ , were drawn from the discrete Uniform (50, 300) distribution. Given the true value of the parameter $\sigma_{\varphi }^{2}$ , a logGamma(5,1) prior was used for $\text{log}(1/\sigma_{\varphi }^{2})$ . Prior specifications for all other parameters remain as discussed in Section 3.2. Five hundred replicate data sets were generated from the model for each of the $\left(r\times n_{A}=9\right)$ simulation settings.

**Figure 3.**
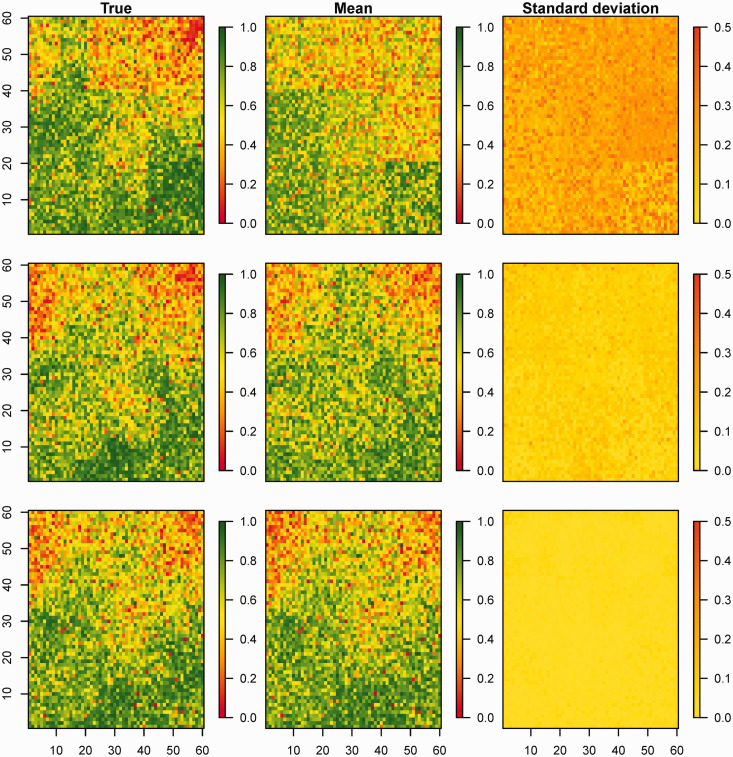
One of the simulated data sets for spatial range *r* = 0.7. Plotted are true simulated probabilities and the corresponding predictions (mean) and their standard deviations for $n_{A}$ = 9 (top), 25 (middle) and 100 (bottom).

To evaluate the predictive performance of the model, we computed the correlations between the observed and predicted probabilities at both the grid and area levels, as well as the root mean square error (RMSE = √∑i=1n(p^i-pi)2/n ) and the actual coverage of the 95% prediction intervals (95% Coverage = 100×∑i=1nI(li≤p^i≤ui)/n ) of the predictions; where $n=n_{A}$ (or $n_{p}$ ), I(.) is an indicator function, and $l_{i}$ and $u_{i}$ are the lower and upper limits of the prediction intervals, respectively. These metrics were averaged over the simulated replicate data sets. Figure 3 shows an example of the simulated data sets for *r* = 0.7. Similar plots for *r* = 0.3 and *r* = 0.5 are shown in online Supplemental Figures S3 and S4. These plots generally show that the model performed well in recovering the simulated images even with $n_{A}=9$ . As expected, better predictions were obtained with increasing values of $n_{A}$ . This is further corroborated by corresponding plots of the observed and predicted probabilities at the grid level shown in online Supplemental Figure S5. Additionally, the standard errors diminish as $n_{A}$ increases in each case as expected.

All the model evaluation criteria reported in Table 1 show that the simulated data at the area level were well estimated by the model regardless of the values of *****r* and $n_{A}$ . Larger RMSE values were obtained at the grid point level compared with the area level values, demonstrating the increased uncertainty associated with the predictions at this level. The minimum correlation between the observed and predicted probabilities was 0.66 while the minimum achieved 95% coverage rate was 80.98%. All three criteria show that improved predictions were obtained with increasing values of the spatial range parameter, *r*. The effect of $n_{A}$ is more pronounced when examining the RMSE and correlation values, both of which indicate better performance with more observations. Overall, these results indicate a good predictive performance by the model.

**Table 1.** Results of the simulation study.

Scale	*r*	RMSE			Correlation			95% Coverage		
		$n_{A}$ = 9	25	100	9	25	100	9	25	100
Area	0.3	0.03	0.03	0.03	0.98	0.99	0.99	92.67	94.80	94.84
	0.5	0.03	0.03	0.03	0.98	0.99	0.99	93.78	95.12	94.29
	0.7	0.03	0.03	0.03	0.98	0.99	0.99	94.00	94.48	95.03

Grid	0.3	0.21	0.12	0.06	0.66	0.88	0.96	84.83	80.98	89.49
	0.5	0.20	0.10	0.05	0.69	0.90	0.97	88.95	86.68	92.12
	0.7	0.18	0.09	0.04	0.76	0.92	0.98	93.47	90.88	93.56

Note: Reported are the RMSEs, correlations and actual coverage of the 95% prediction intervals averaged over the 500 replicate data sets.

## 5 Mapping the coverage of measles and DTP1, 2 and 3 vaccinations in Afghanistan and Pakistan

We now apply the proposed methodology to predict vaccination coverage in the study countries at 5×5 km^2^ resolution using the data sets discussed and presented in Section 2. Separate models were fitted for measles and each of the three doses of DTP, with the same set of country-specific covariates used each time. The same prior specifications as provided in Section 3.2 were used in all the analyses. To reduce the variance in the covariates and encourage symmetry, these were log-transformed. Similar approaches were also used in Utazi et al.^12^

The resulting posterior inference summary is provided in Table 2 for measles and DTP3 and online Supplemental Table S1 for DTP1 and DTP2. In all the fitted models, vaccination coverage had a positive relationship with population density and was negatively correlated with travel time, corroborating findings in previous research.^12,36^ However, due to the large standard errors associated with the regression coefficients, none of the estimated relationships were significant in any of the models as the 95% credible intervals in Table 2 reveal. This may be due to lack of vaccination coverage data at the grid level to support the estimation of these parameters or as a result of collinearity (the covariates were uncorrelated at the grid level but moderately negatively correlated at the area level in both countries), although the latter is not a major concern here as inference is geared towards prediction. For all vaccination types, significant correlations in vaccination coverage were detected between the subnational areas in both countries through the spatial random effect, φ. This is evidenced by the estimates of ρ ( 0.41≤ρ^≤0.61 ), which are significant in all the models. The estimated spatial ranges for ***η*** (given in decimal degrees in Table 2 and online Supplemental Table S1), correspond to distances of 167, 266, 185 and 272 km in Afghanistan (maximum distance = 1891 km) and 982, 1304, 1140 and 1217 km in Pakistan (maximum distance = 2415 km), for measles and DTP1, 2 and 3, respectively. These show much higher levels of spatial dependence in vaccination coverage in Pakistan than Afghanistan; although we note that the sizes and spatial arrangements of the input areal units in each country may have influenced these estimates. Also, spatial correlation in measles vaccination coverage appears to be lower than that of the coverage of the doses of DTP in both countries. The estimates of κ are not reported as these can be obtained from r^ . In general, these parameter estimates show that significant spatial dependence was estimated at both the areal and grid levels in all the models through φ and ***η***, respectively. The different models/covariance structures assumed for these random effects, however, implies that their relative contributions to explaining the variability in the data cannot be determined via these parameters.

**Table 2.** Posterior estimates of the parameters of the fitted models for measles and DTP 3.

	Afghanistan			Pakistan		
Parameter	Mean	SD	95% CI	Mean	SD	95% CI
	Measles					
Intercept	3.1793	4.5037	(−5.4028, 12.2947)	2.5986	2.7924	(−2.6947, 8.5872)
log(Pop. density)	0.2577	0.2832	(−0.2984, 0.8206)	0.1059	0.2280	(−0.3626, 0.5509)
log(Travel time)	−0.3751	0.7481	(−1.8861, 1.0562)	−0.4364	0.4501	(−1.3829, 0.4273)
$\sigma_{\eta }^{2}$	3.7918	0.3996	(3.1141, 4.6786)	1.0500	1.2898	(0.1160, 4.5734)
*r*	1.5060	0.3163	(0.9838, 2.2141)	8.8329	5.9609	(2.0180, 24.6937)
$\sigma_{\varphi }^{2}$	0.0245	0.0308	(0.0022, 0.0995)	0.0286	0.0495	(0.0014, 0.1370)
*ρ*	0.5002	0.2297	(0.0873, 0.8982)	0.5089	0.2705	(0.0476, 0.9473)

	DTP 3					
Intercept	3.1563	4.6732	(−5.6294, 12.7946)	1.7645	3.5386	(−5.2044, 9.0775)
log(Pop. density)	0.3047	0.2852	(−0.2545, 0.8757)	0.2403	0.2780	(−0.3120, 0.8060)
log(Travel time)	−0.3756	0.7713	(−1.9550, 1.0851)	−0.3075	0.5521	(−1.4085, 0.8086)
$\sigma_{\eta }^{2}$	4.7797	0.5425	(3.8275, 5.9432)	1.9646	2.1673	(0.2813, 7.9210)
*r*	2.4479	0.4991	(1.6366, 3.5893)	10.9479	6.9466	(2.6441, 29.1611)
$\sigma_{\varphi }^{2}$	0.0303	0.0597	(0.0014, 0.1549)	0.0228	0.0368	(0.0009, 0.1033)
*ρ*	0.4670	0.1960	(0.1154, 0.8328)	0.4966	0.2664	(0.0535, 0.9462)

The estimates of vaccination coverage for areas with missing data as identified in Section 2 are reported in online Supplemental Table S3. In all cases, the estimated areal values for the areas with observations had very high correlations (>0.97) with the observed values. Also, the RMSEs of the areal predictions for Afghanistan were <0.03 while those of Pakistan were <0.04. These statistics confirm the accuracy of the predictions for the missing areas; however, it should be noted that the model does not account for other conditions, such as security issues, which could affect vaccination coverage levels in these areas.

**Figure 4.**
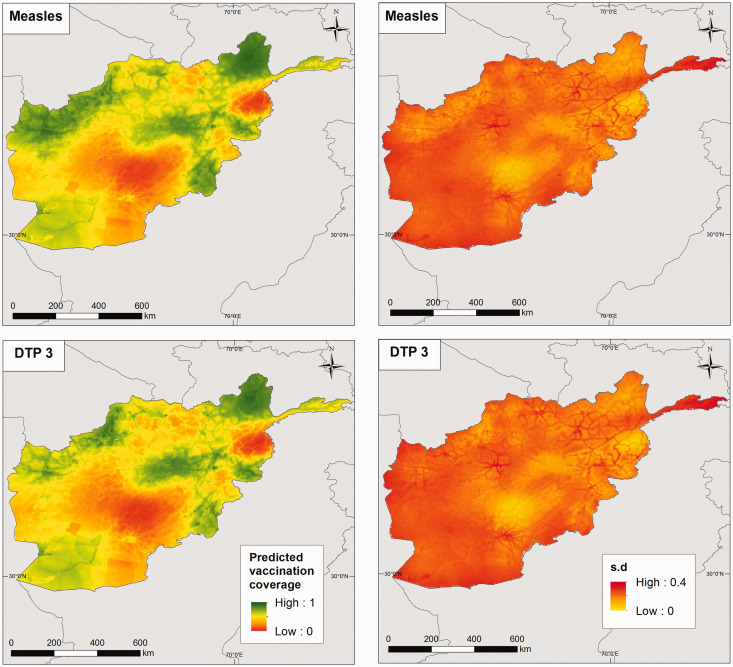
Predicted measles and DTP3 vaccination coverage at 5 × 5 km^2^ (left panel) in children aged 12–23 months for Afghanistan in 2015, with the associated standard deviation maps (right panel).

**Figure 5.**
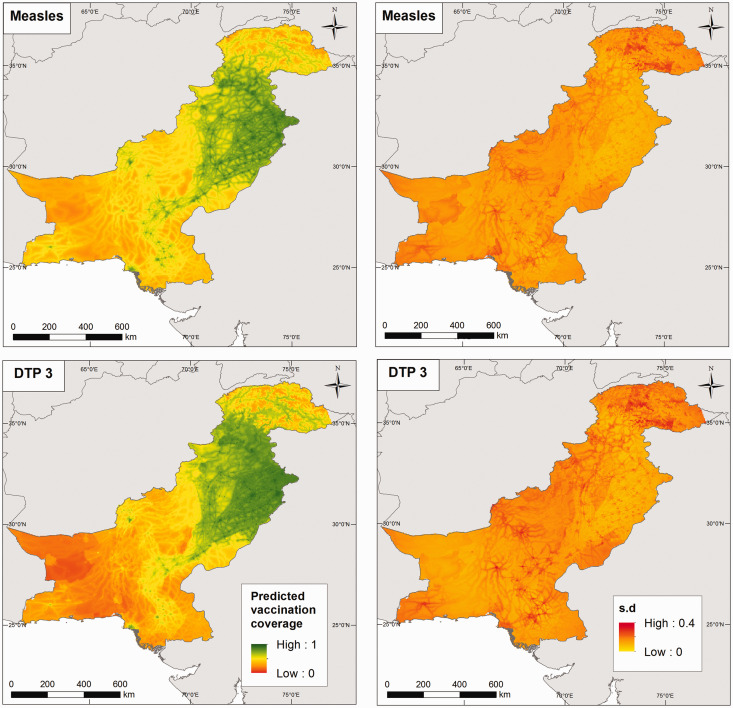
Predicted measles and DTP3 vaccination coverage at 5 × 5 km^2^ (left panel) in children aged 12–23 months for Pakistan in 2013, with the associated standard deviation maps (right panel).

The predicted vaccination coverage levels at 5×5 km^2^ for both countries are mapped in Figure 4 and online Supplemental Figure S6 for Afghanistan, and Figure 5 and online Supplemental Figure S7 for Pakistan. These maps suggest significant fine-scale heterogeneities in vaccination coverage within each country, which are not apparent when examining the areal data shown in Figure 1 and online Supplemental Figure S1. Similar trends are seen in coverage levels in both measles and DTP in each country especially with respect to the lowest coverage areas. Additionally, vaccination coverage appears to decrease as expected with higher doses of DTP. Some patterns mirroring the effects of access/remoteness (see online Supplemental Figure S2) on vaccination coverage are also visible in these maps. In Afghanistan, the lowest coverage areas for both measles and DTP are concentrated in the central and south-eastern parts of the country and the province of Nuristan. Although for DTP1, higher coverage levels were obtained in the south-eastern axis and other areas compared to other vaccines. The associated standard deviation maps shown on the right panel of Figure 4 indicate that the predictions generally had low uncertainties. In particular, these show that some low coverage areas were estimated with low uncertainty. In Pakistan, areas of high vaccination coverage predominantly occurred in the province of Punjab and parts of Khyber Pakhtunkhwa and Sindh, in mostly high-population density areas. The standard deviation maps show that the predictions were obtained with higher precision compared with Afghanistan.

**Figure 6.**
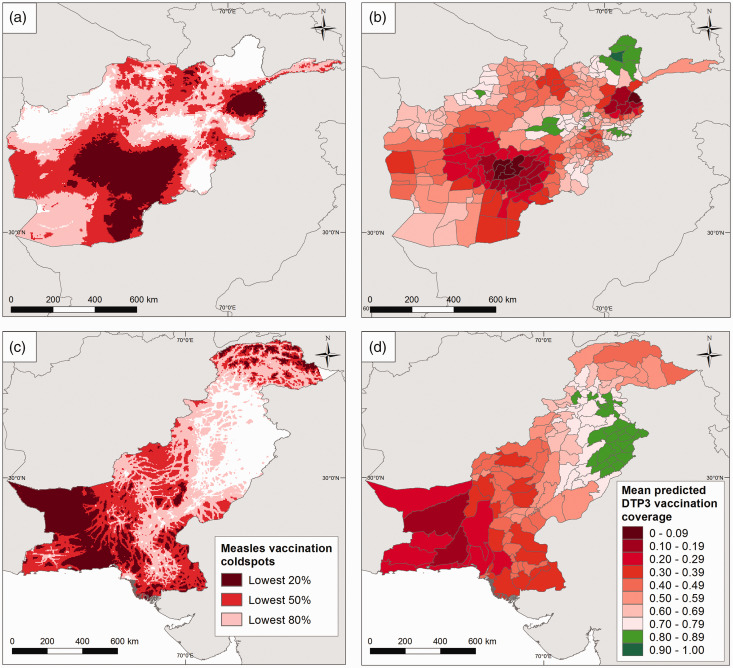
Maps of Afghanistan (top) and Pakistan (bottom) showing: (a and c) coldspot areas for measles vaccination defined as the lowest 20%, lowest 50% and lowest 80% coverage areas and (b and d) the districts attaining the WHO Global Vaccine Action Plan (GVAP) threshold of 80% coverage (in green colour) with DTP3 vaccination for Afghanistan in 2015 and Pakistan in 2013.

Of significant epidemiological interest in the evaluation of measles vaccination coverage is the identification of ‘coldspots’.^11^ Coldspots are low coverage areas that foster and accelerate disease circulation, and should thus be designated as priority areas when planning immunization and disease elimination programmes. In Figure 6 (right panel), we define coldspot areas using flexible thresholds pertaining to the overall coverage levels within each country. These are: the lowest 20%, lowest 50% and lowest 80% coverage areas, corresponding to cutpoints of 0.33, 0.55 and 0.70 for Afghanistan, and 0.35, 0.47 and 0.67 for Pakistan, respectively. These maps show that significantly large areas of both countries, as explained previously, are coldspots of low vaccination coverage, especially when considering the 80% threshold within each country. However, for effective planning using these maps, the identified coldspots must be combined with maps of population estimates to determine whether significant numbers of unvaccinated children exist in these areas (see Takahashi et al.^11^).

The WHO Global Vaccine Action Plan (GVAP) has set a target of attaining 80% coverage with all vaccines in all countries by 2020.^37^ We illustrate the evaluation of progress towards this target using DTP3 vaccination coverage maps. (Given that this target was set at the district level, this evaluation would not have been possible using the areal data in Figure 1.) For each country, the 5 × 5 km^2^ predictions were aggregated to the district level by averaging the predictions over the grid cells within each district – this is a standard approach used in geostatistics. The maps in Figure 6 (right panel) show that for Afghanistan in 2015 and Pakistan in 2013 only 8% and 19% of districts had attained these targets, respectively. In Pakistan, in particular, these districts are mostly located in the province of Punjab, matching findings elsewhere.^38^

## 6 Predictive performance comparisons with high-resolution maps obtained using geolocated cluster level data

High-resolution maps of vaccination coverage have been produced in previous research using geolocated survey data.^11,12^ Here, we undertake additional data analyses to compare maps of measles vaccination coverage in children aged 12–23 months produced using the proposed methodology which utilizes weighted areal data (see, e.g. Section 2) with equivalent maps obtained using geolocated cluster level data, in settings where both data sets were available. The countries considered in this analysis were Cambodia, Mozambique and Nigeria, for which the most recent DHS surveys were conducted in 2014, 2011 and 2013, respectively. For the analysis with areal data, we used the most detailed administrative (admin) level at which the surveys were deemed representative. These were the 19 regions (as groups of admin level 1 areas) of Cambodia, the 11 admin level 1 areas of Mozambique and the 37 states (including the capital) of Nigeria. Combining weighted vaccination coverage data for these areas with some covariate data identified in a previous modelling exercise in Utazi et al.^12^ (see online Supplemental materials for details) model (1) was applied to predict vaccination coverage on a 5×5 km^2^ grid for each of the three countries.

To obtain vaccination coverage maps using geolocated cluster level data, we modified model (1) by replacing the areal data with cluster level data and removing the areal random effect, φ, yielding

(4) Yi∼Binomial(Ni,pi),i=1,…,nc,nc+1,…,nc+nplogit(pi)=xi∼'β+η(si),   i=1,…,nclogit(pi)=xi'β+η(si),   i=nc+1,…,nc+np

for $n_{c}$ cluster (or observation) locations (with known GPS coordinates) and $n_{p}$ prediction grid points; with the ${\overset{∼}{x}}_{i}^{'}$ 's representing covariate values for the cluster locations which also adjust for the random displacement of the locations; see Utazi et al.^12^ for details. All other terms in equation (4) are the same as before, with appropriate changes to the definition of the spatial random effect, η. The geostatistical model in equation (4) was fitted using the INLA-SPDE approach. The same priors as discussed in Section 3.2 and covariates, as in the analysis using areal data, were used.

The resulting coverage maps and associated uncertainties are shown in online Supplemental Figures S8 to S10 for all three countries, alongside the differences between the two approaches. A visual inspection of the maps shows that while the two approaches are mostly similar in predicting high and low coverage areas together with the associated uncertainties, some differences are apparent in some areas. However, the average differences of 0.04 (interquartile range (IQR) = 0.08), 0.05 (IQR = 0.09) and 0.05 (IQR = 0.13) for Cambodia, Mozambique and Nigeria, respectively, as reported in online Supplemental Table S5 indicate that, in general, strong similarities exist between the two approaches.

**Figure 7.**
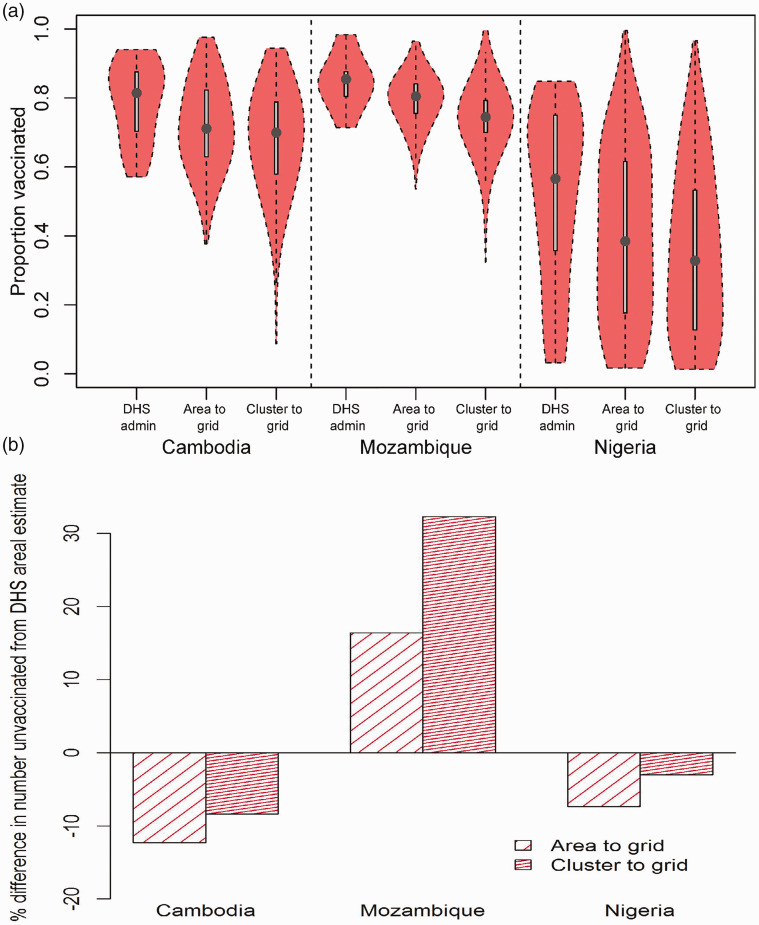
(a) Distributions of proportions of children aged 12–23 months vaccinated against measles and (b) percentage differences in national estimates of numbers of under 5 year olds unvaccinated between DHS admin estimates and each of the 5 × 5 km^2^ estimates from area-to-grid and cluster-to-grid approaches. Vaccination coverage in children aged 12–23 months was used as a proxy for coverage levels in under 5s in (b).

In Figure 7(a), we compare for each country, the distributions of the 5×5 km^2^ predictions obtained using both approaches (i.e. area-to-grid and cluster-to-grid) and DHS admin estimates. It is evident that both gridded maps unmask more heterogeneities and coldspots of low vaccination coverage that are often missed by large area summaries such as DHS areal estimates. This is corroborated by the lower mean values and greater variabilities around the means estimated through the maps compared to DHS estimates. The contrasts between the gridded maps and DHS estimates are also reflected in the differences in numbers of unvaccinated children (online Supplemental Table S5) estimated through integrating vaccination coverage estimates (used as a proxy for coverage in under 5s) from all three approaches with matching (at area and grid levels) United Nations-adjusted estimates of children aged under 5 years obtained from the WorldPop database (www.worldpop.org.uk). Figure 7(b) shows that there is an agreement between the gridded maps in estimating higher or lower numbers compared to DHS estimates, with differences of up to 32% seen in Mozambique. Interestingly, in Cambodia and Nigeria, lower numbers of unvaccinated children were estimated through using the gridded maps compared to using DHS areal estimates. These differences are related to the distribution of urban and rural areas and areas of high-population density in these countries which are better accounted for by the gridded maps. In summary, these comparisons demonstrate that the high-resolution maps produced using the proposed methodology are highly comparable to those obtained through using conventional cluster-to-grid approaches.

## 7 Discussion

Spatially detailed data is key in the era of the SDGs with the central focus of ‘leaving no one behind’ and the push for precision public health^39^ as a strategy for achieving disease elimination and allocating scarce resources. In resource poor settings, high-resolution maps of key health and development metrics are increasingly derived from geolocated cluster level survey data through spatial interpolation methods. Here, we have developed a methodology for producing high-resolution maps from areal survey data where geolocated cluster level information is unavailable, focusing on binomial responses arising from the application to vaccination coverage mapping. The areas and the high-resolution grids were linked in the proposed model using the regression component and both of the spatial random effects in a hierarchical Bayesian framework. The INLA-SPDE approach provided a fast and computationally efficient method for implementing the model. A simulation experiment demonstrated the predictive ability, and high-resolution mapping of measles and DTP vaccination, the applicability of the methodology. The value of the high-resolution maps of vaccination coverage produced is illustrated through the identification of coldspots of low coverage and an assessment of progress toward vaccination targets. These output maps, when combined with population estimates, as demonstrated in Section 6, can be used to generate estimates of numbers of unvaccinated children, particularly those living in coldspot areas, as well as estimates of numbers children who have received the first dose of a vaccine but not the latter dose(s) to help with the planning and implementation of vaccination programmes, and other disease eradication and health improvement efforts (see Takahashi et al.^11^).

While geolocated cluster-level survey data are ideal for geostatistical mapping of development indicators due to their level of spatial detail and availability of ready-to-use methodological approaches, there are however some advantages to modelling using area level data. First, areal summaries are unaffected by the displacement of survey cluster coordinates often carried out to protect respondents' confidentiality.^2^ Secondly, most surveys such as those undertaken as part of the DHS program are designed to be representative at the area level. This facilitates the application of sampling weights to areal summaries to account for the survey design. Currently, we are not aware of any appropriate technique for accounting for the survey design when producing high-resolution maps from cluster-level data using geostatistical approaches, although it has been noted that these may not substantially change the predicted maps.^40^ Thirdly, areal data being aggregates of cluster-level data are, in principle, unlikely to be affected by sample size issues often encountered when using cluster-level data in binomial models.^12^ Most importantly, the comparisons undertaken in Section 6 have shown that maps produced using areal data through the proposed methodology are highly comparable to those produced using cluster level data, notwithstanding the fact that weighted areal data were used in these comparisons.

There are limitations in this work in terms of predictive accuracy relating to the number and size of the input areal units. In the data sets analysed, this limitation is particularly evident in Pakistan where observed vaccination coverage levels correspond to large, sparse administrative units. Since predictive accuracy (see Section 4) increases as $n_{A}$ increases (yielding smaller-sized areas), the design of future DHS surveys for this country using a more disaggregated administrative level would be an effective, though costly and logistically challenging, solution to this problem. Alternatively, other surveys such as the Pakistan Social and Living Standard Measurement Survey^36^ which provide data at both the provincial and district levels could be considered. Furthermore, the proposed methodology uses an estimation approach which generates predictions at the grid level during model-fitting, as against a prediction approach in which model-fitting and prediction operations could be separated; thus facilitating the implementation of parallel computing to achieve further savings in computational time during prediction. This estimation approach meant that to obtain predictions at 5 × 5 km^2^ for Pakistan, for example, an additional computing memory greater than what was available on a 16 GB RAM machine was required due to the large number of prediction grids. Thus, despite being implemented using the fast INLA-SPDE method, higher computing power will be needed in applications involving much larger spatial domains or multiple countries should a similar spatial resolution be required.

In the vaccination coverage mapping application, the geospatial covariates used did not include some variables that are known to influence access to and acceptance of vaccines such as health facility access,^41^ maternal literacy^38^ and vaccine stocks. The inclusion of these variables in the analyses, where their spatial surfaces exist, could further improve the predicted maps. In addition, the selected covariates used in the applications were based on previous studies as pointed out in Section 2. In situations where there is no prior information on the relationships between the variable of interest and available covariates, the observed areal data could be used to perform covariate selection. The no-covariate case is not of interest in this work as a previous study^18^ has shown that predictive performance decreases in this case. On a related note, the specification of uniform regression coefficients for all the areas in the model has the potential to obscure area-specific variations in the relationships between the covariates and vaccination coverage. For example, an overall positive relationship between vaccination coverage and population density implies that anti-vaccine populations cannot be accounted for by the model. Lastly, in the simulation study in Section 4, the effects of different values of $n_{A}$ and the spatial range parameter, *r*, investigated were deemed to have direct practical implications in this work. However, evaluating the effects of varying values of other parameters such as the autocorrelation parameter, ρ, the regression coefficients, ***β***, and the scale parameters, $\sigma_{\eta }^{2}$ and $\sigma_{\varphi }^{2}$ , may shed more light on other aspects of the predictive performance of the model.

We envisage future work in several directions. Here, we focused on binomial responses. However, the methodology is easily extensible to other outcome distributions and can be used to model many other health and development indicators. Vaccination coverage is known to vary by age,^11,12^ with age-specific coverage levels providing valuable information and insights. Although the 12 to 23-month age group analysed here is a standard age group used for evaluating the effectiveness of vaccination programmes, we plan to explore coverage mapping for other age groups of under 5s in the countries studied. Extensions to other childhood vaccinations and more countries without geolocated survey data will also be considered. Lastly, as demonstrated in Moraga et al.,^18^ it is straightforward to introduce point-level data in the proposed methodology (by combining equations (1) and (4)) to form a fusion model. This modelling framework could be used to adjust for the survey design (through the areal data) in geostatistical mapping of development indicators using geolocated survey data.

## Supplemental Material

Supplemental material for A spatial regression model for the disaggregation of areal unit based data to high-resolution grids with application to vaccination coverage mapping Supplemental material for A spatial regression model for the disaggregation of areal unit based data to high-resolution grids with application to vaccination coverage mapping by CE Utazi, J Thorley, VA Alegana, MJ Ferrari, K Nilsen, S Takahashi, CJE Metcalf, J Lessler and AJ Tatem in Statistical Methods in Medical Research
