# Supplementary material for: A spatial regression model for the disaggregation of areal unit based data to high-resolution grids with application to vaccination coverage mapping
Source: Stat Methods Med Res. 2018 Sep 19;28(10-11):3226–41. doi: 10.1177/0962280218797362 (PMC6745613; doi:10.1177/0962280218797362)
Supplement: Supplemental material for A spatial regression model for the disaggregation of areal unit based data to high-resolution grids with application to vaccination coverage mapping [file Supplemental_material.pdf]

A spatial regression model for the disaggregation of areal unit based data to high resolution grids with application to vaccination coverage mapping

Utazi CE, Thorley J, Alegana VA, Ferrari MJ, Nilsen K, Takahashi S, Metcalf CJE, Lessler J and Tatem AJ

Supplemental materials

This document contains additional figures and tables, details of the analysis in Section 6 and the R code for implementing the proposed methodology, all of which are referenced in the main paper.

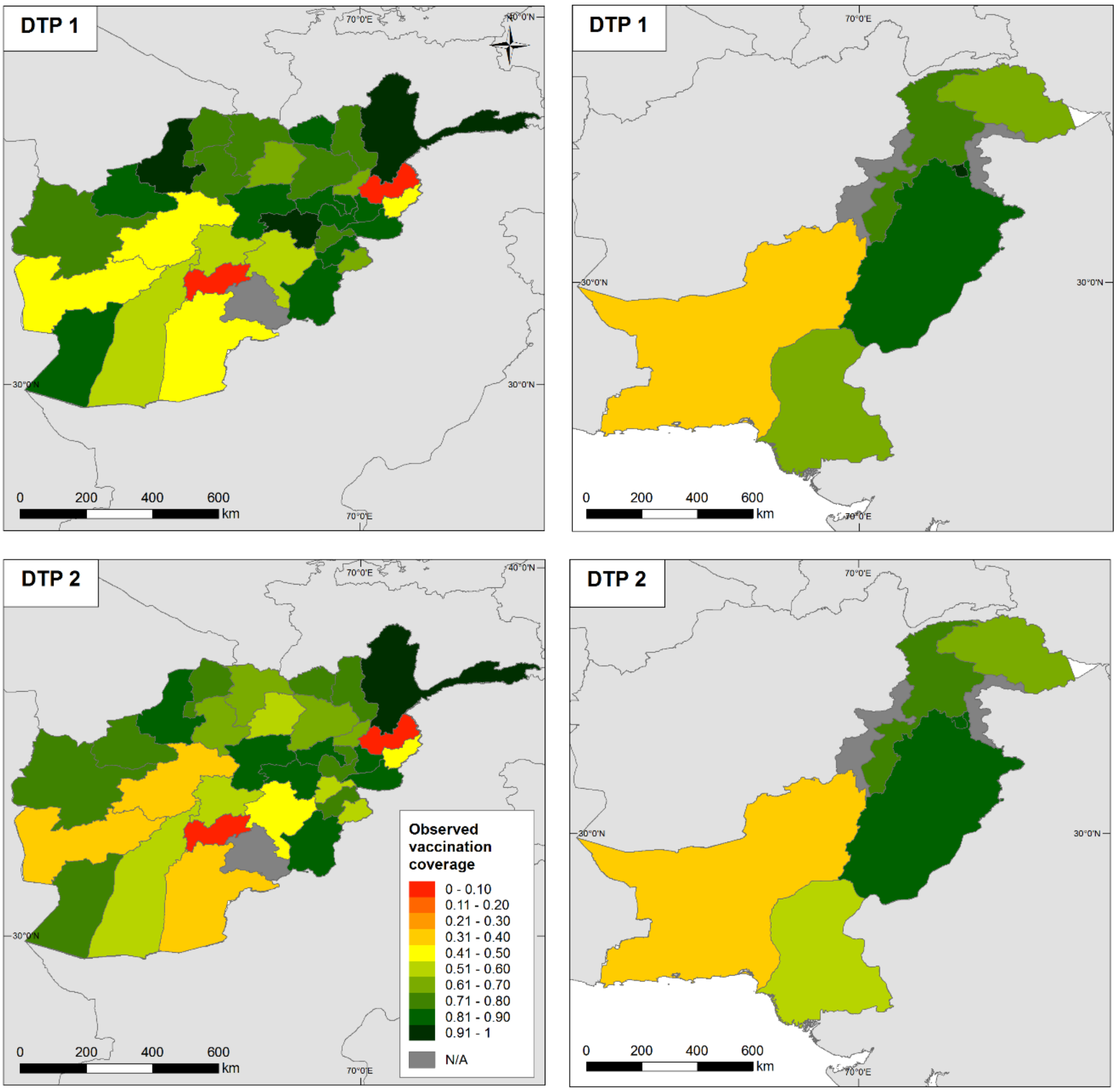

Figure S1: Maps of observed DTP1 and DTP2 vaccination coverage for Afghanistan in 2015 (left panel) and Pakistan in 2013 (right panel)

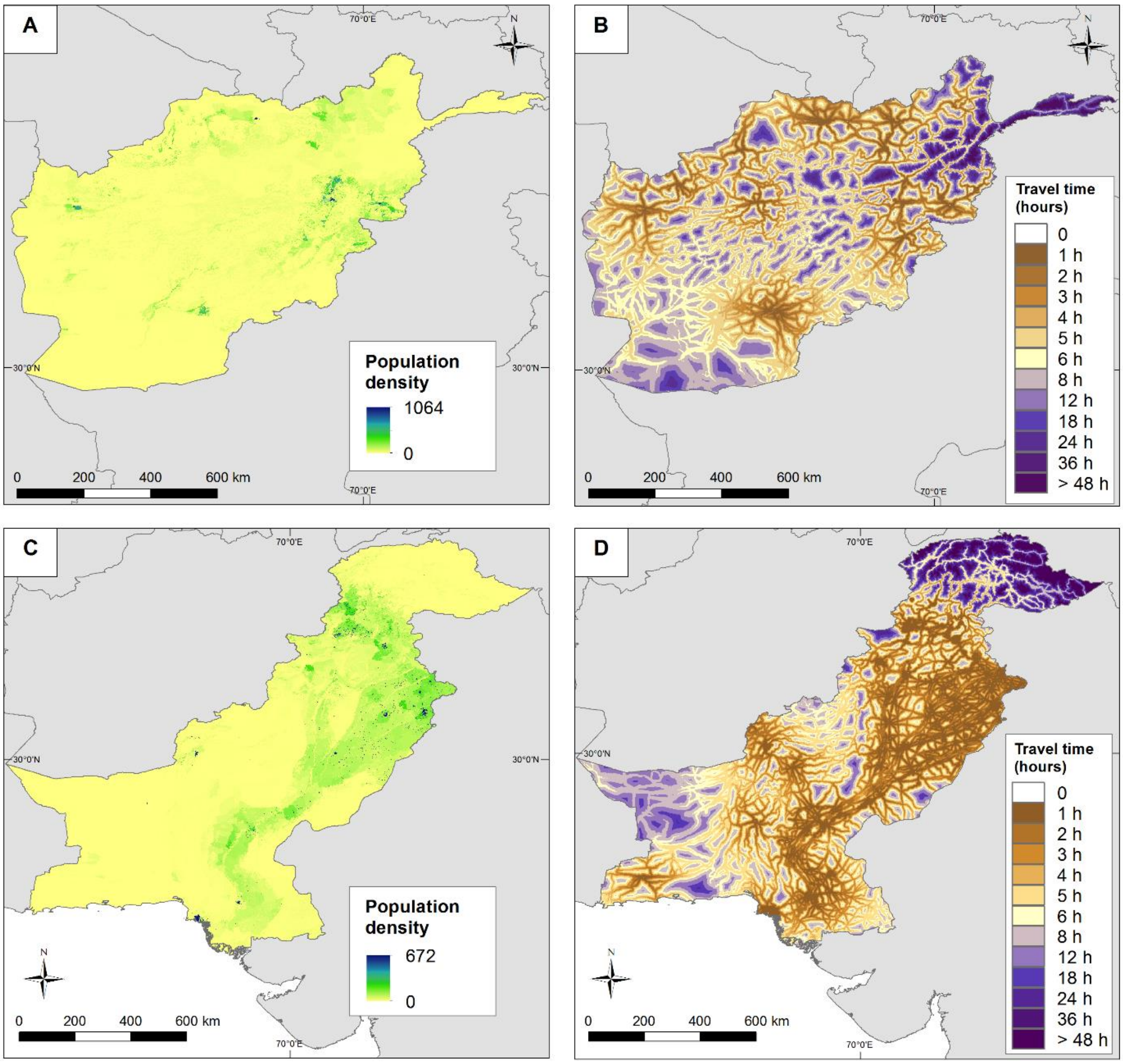

Figure S2: Maps of covariate data used in the analysis for Afghanistan (top row) and Pakistan (bottom row)

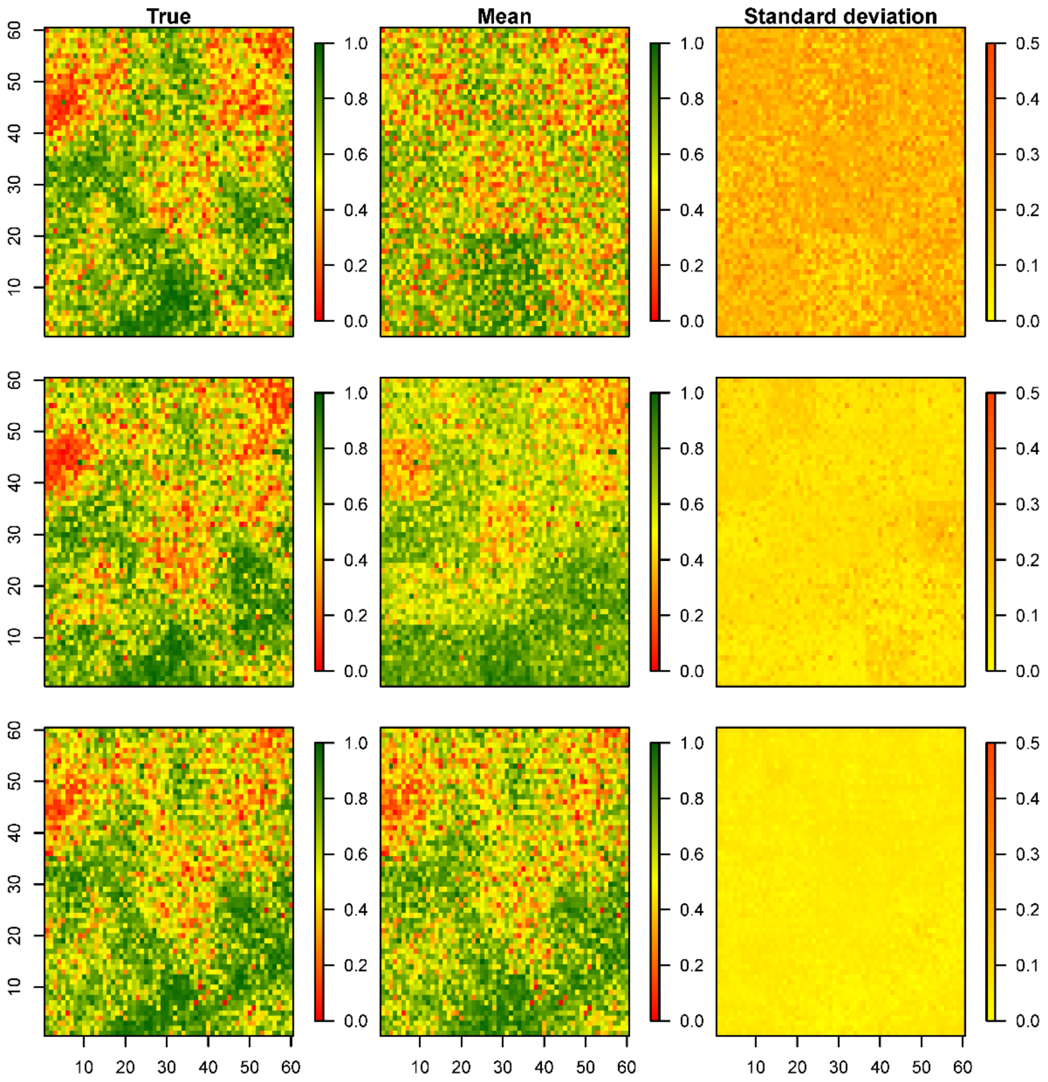

Figure S3: One of the simulated data sets for spatial range $r = 0.3$. Plotted are true simulated probabilities and the corresponding predictions (mean) and their standard deviations for $n_{\mathcal{A}}$ =9 (top), 25 (middle) and 100 (bottom).

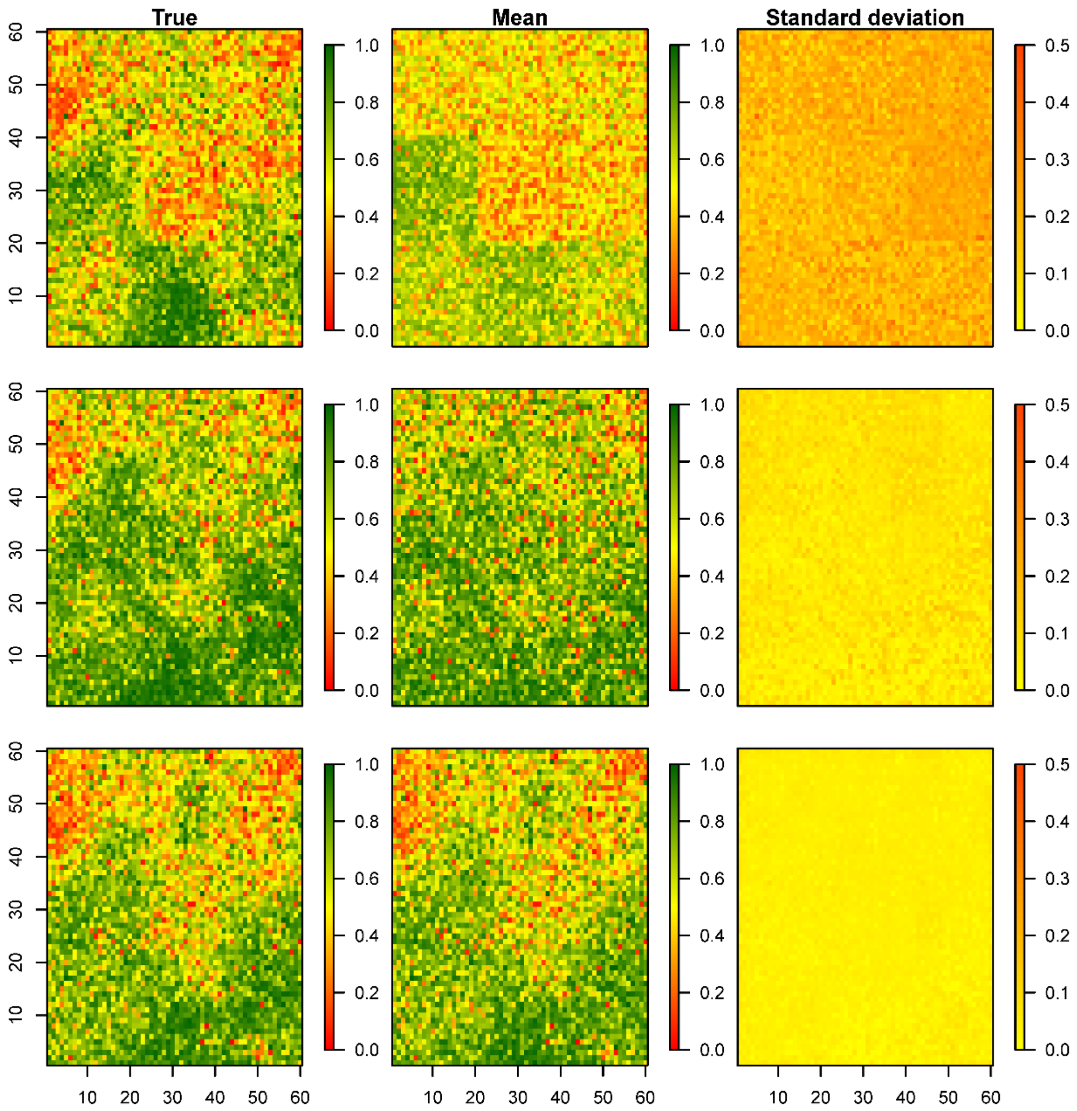

Figure S4: One of the simulated data sets for spatial range $r = 0.5$. Plotted are true simulated probabilities and the corresponding predictions (mean) and their standard deviations for $n_{\mathcal{A}}$ =9 (top), 25 (middle) and 100 (bottom). Figure 3 Plots of observed vs predicted probabilities at the grid level

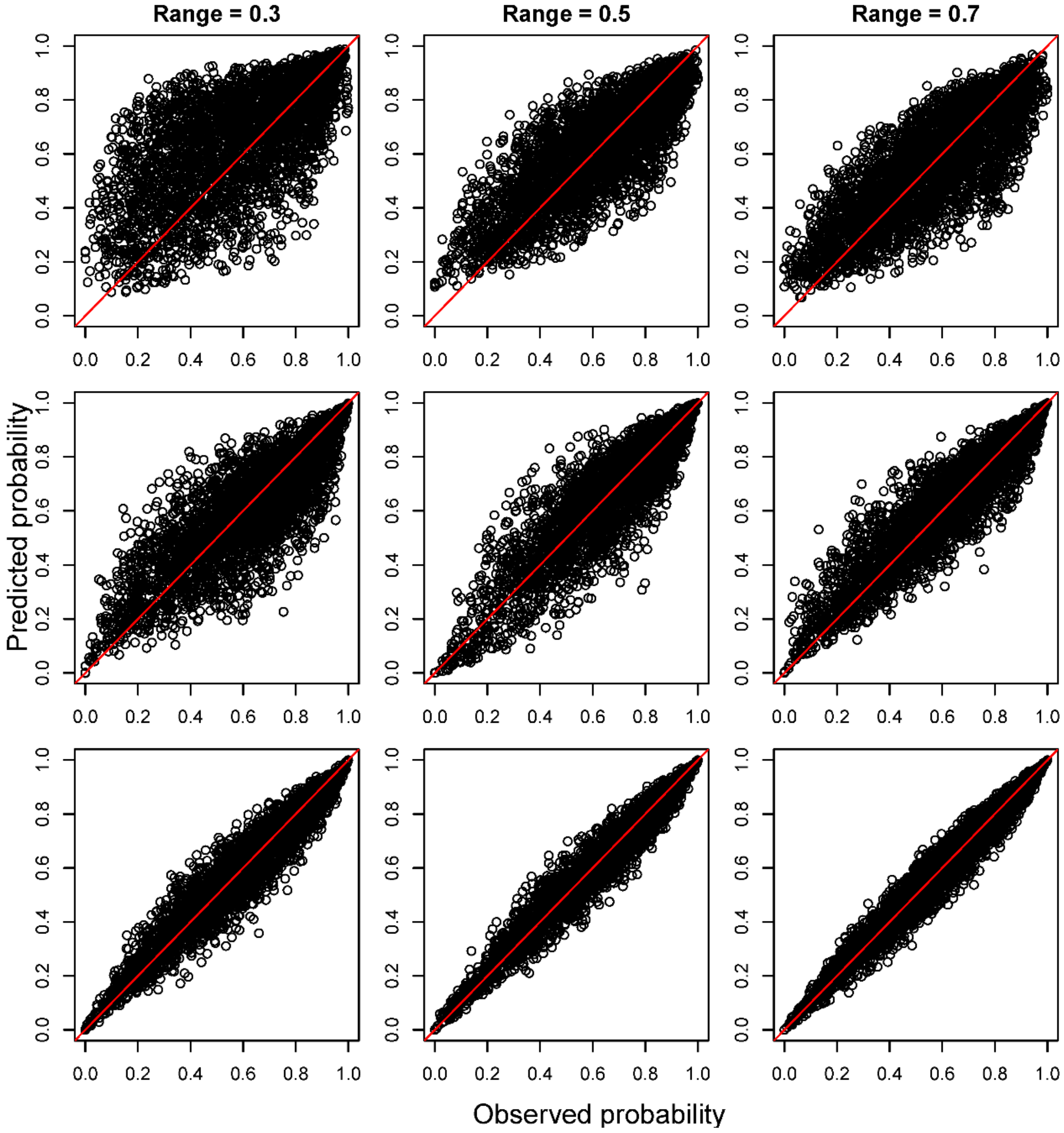

Figure S5: Plots of observed vs predicted values for some of the simulation datasets as described in Section 4 in the main paper: Top ($n_{\mathcal{A}}$ = 9), middle ($n_{\mathcal{A}}$ = 25), and bottom $n_{\mathcal{A}}$ = 100.

Table S1: Posterior estimates of the parameters of the fitted models for DTP1 and DTP2

	Afghanistan			Pakistan		
Parameter	Mean	SD	95% CI	Mean	SD	95% CI
	DTP 1					
Intercept	8.0859	6.2315	(-3.4445, 20.4346)	3.3620	3.4890	(-3.3731, 10.7064)
log(Pop. density)	0.0758	0.3474	(-0.6054, 0.7590)	0.3237	0.2744	(-0.2186, 0.8762)
log(Travel time)	-1.0979	1.0303	(-3.1378, 0.8152)	-0.4717	0.5559	(-1.6048, 0.6242)
σ_η^2	7.5698	2.7097	(3.6454, 14.2256)	1.9992	2.6069	(0.1757, 9.0936)
r	2.3910	0.5758	(1.3930, 3.6422)	11.7280	7.9953	(2.7551, 33.0398)
σ_ϕ^2	0.0322	0.0712	(0.0012, 0.1711)	0.0275	0.0496	(0.0011, 0.1342)
ρ	0.4188	0.0585	(0.3016, 0.5307)	0.5046	0.2644	(0.0572, 0.9454)
	DTP 2					
Intercept	4.1043	5.0544	(-5.5975, 14.3166)	1.5028	3.1388	(-4.6653, 8.0312)
log(Pop. density)	0.2978	0.3437	(-0.3794, 0.9786)	0.3151	0.2546	(-0.2042, 0.8155)
log(Travel time)	-0.4678	0.8477	(-2.1776, 1.1651)	-0.1931	0.4975	(-1.1978, 0.7993)
σ_η^2	5.3880	0.2117	(4.9440, 5.7809)	1.3869	1.6764	(0.1561, 5.9771)
r	1.6649	0.0687	(1.5244, 1.7954)	10.2501	6.9608	(2.3390, 28.8157)
σ_ϕ^2	0.0122	0.0035	(0.0083, 0.0221)	0.0297	0.0543	(0.0011, 0.1455)
ρ	0.6138	0.0543	(0.5307, 0.7457)	0.5019	0.2677	(0.0537, 0.9486)

Table S2: DIC values of the fitted models

	Afghanistan		Pakistan	
	Pd	DIC	Pd	DIC
Measles	30.78	234.39	5.07	40.87
DTP1	30.70	225.50	5.08	38.71
DTP2	31.14	230.72	5.12	40.06
DTP3	30.75	236.35	5.16	40.04

Table S3: Estimates of vaccination coverage for the regions of Zabul in Afghanistan and Azad Jammu and Kashmir and FATA in Pakistan

	Zabul		
	Mean	SD	95% CI
Measles	0.1796	0.1463	(0.0175, 0.5712)
DTP1	0.2663	0.1984	(0.0227, 0.7507)
DTP2	0.2099	0.1836	(0.0127, 0.6962)
DTP3	0.1656	0.1391	(0.0154, 0.5434)
	Azad Jammu and Kashmir		
Measles	0.6550	0.0904	(0.4788, 0.8394)
DTP1	0.8295	0.0690	(0.6732, 0.9492)
DTP2	0.7723	0.0765	(0.5982, 0.9069)
DTP3	0.7461	0.0859	(0.5510, 0.8976)
	FATA		
Measles	0.5449	0.1100	(0.2956, 0.7557)

DTP1	0.6757	0.1204	(0.3692, 0.8670)
DTP2	0.6406	0.1125	(0.3705, 0.8375)
DTP3	0.6009	0.1256	(0.3076, 0.8261)

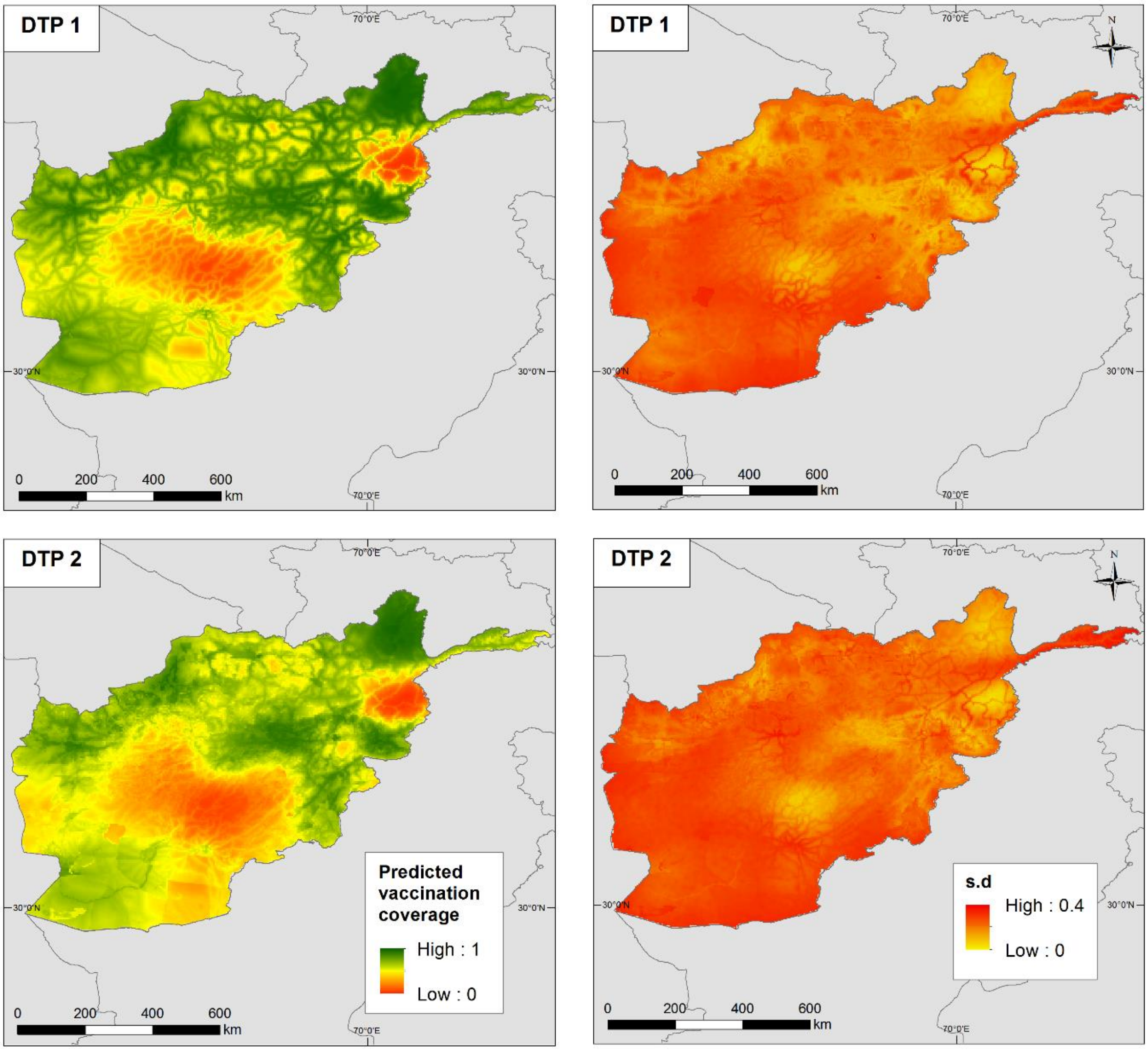

Figure S6: Predicted DTP1 and DTP2 vaccination coverage at 5×5 km (left panel) in children aged 12-23 months for Afghanistan in 2015, with the associated standard deviation maps (right panel).

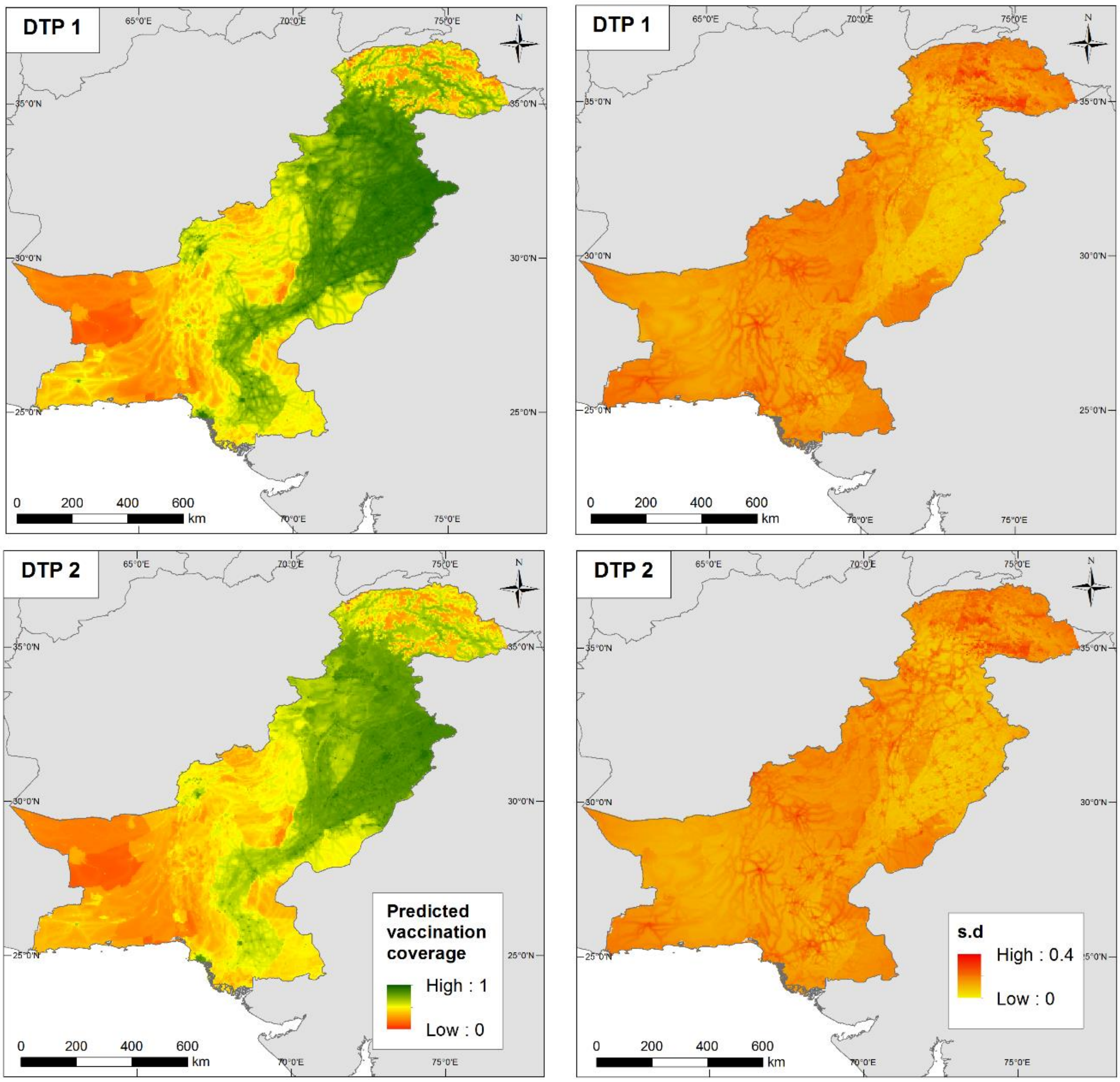

Figure S7: Predicted DTP1 and DTP2 vaccination coverage at 5×5 km (left panel) in children aged 12-23 months for Pakistan in 2013, with the associated standard deviation maps (right panel).

Details of predictive performance comparisons with high resolution maps obtained using geo-located cluster-level data

The covariates included in the area-to-grid and cluster-to-grid analyses were: travel time and population density for Cambodia, travel time and MODIS net primary production for Mozambique, and travel time and poverty for Nigeria; all of which were identified as important predictors of vaccination coverage in these countries in a previous modelling exercise in Utazi et al[1].

Figures S8-10 display the coverage maps and associated uncertainties using the proposed model (area-to-grid) and cluster-to-grid approaches, as well as the differences between these approaches. Other tables referenced in Section 6 are given as follows.

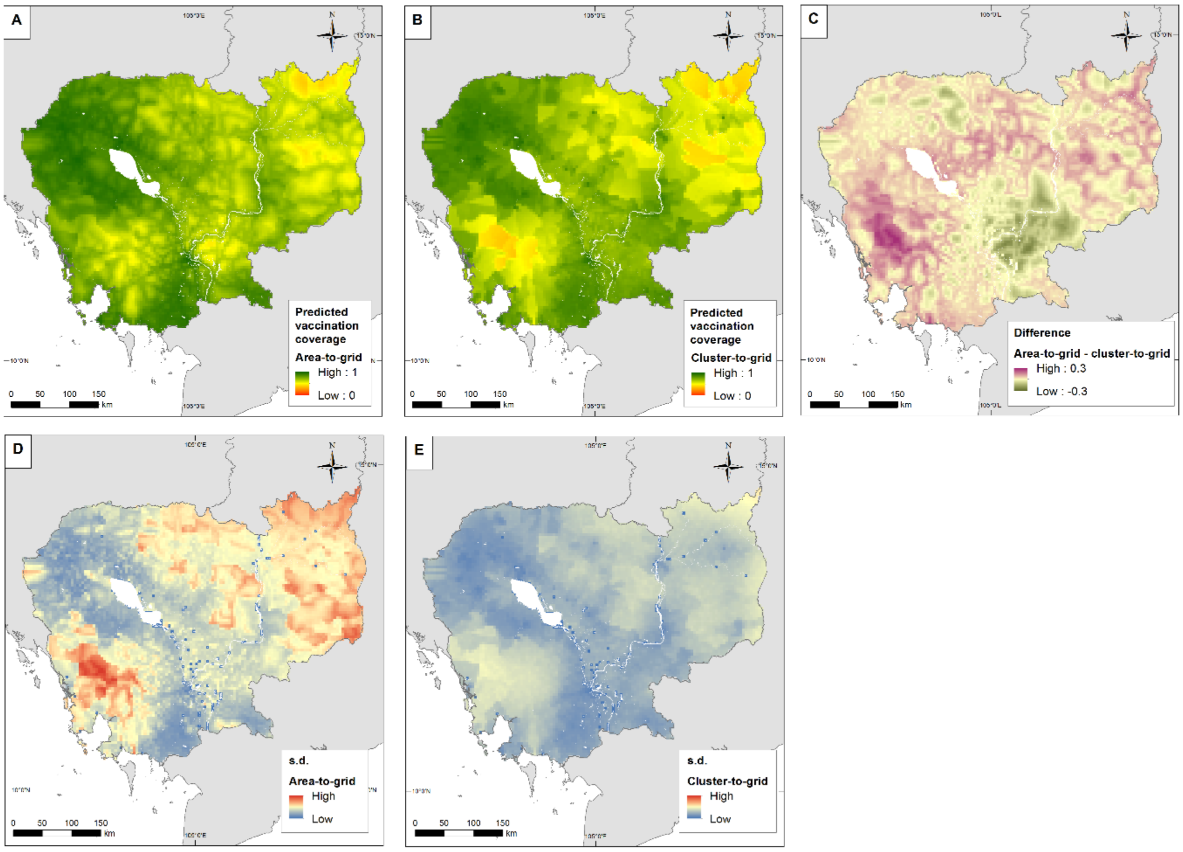

Figure S8: Predicted measles vaccination coverage and associated standard deviations at 5×5 km through using area-to-grid and cluster-to-grid approaches for Cambodia in 2014. The differences between the two approaches are shown in panel (C).

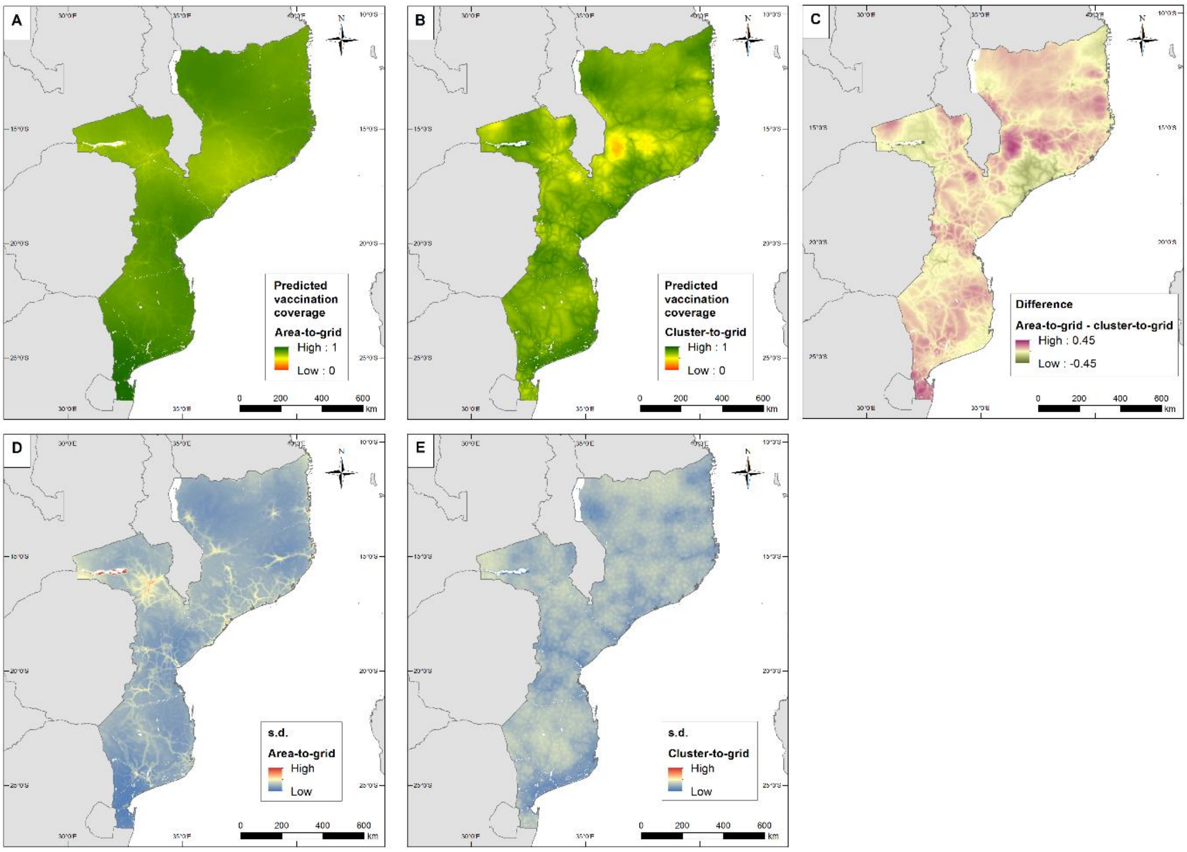

Figure S9: Predicted measles vaccination coverage and associated standard deviations at 5×5 km through using area-to-grid and cluster-to-grid approaches for Mozambique in 2011. The differences between the two approaches are shown in panel (C).

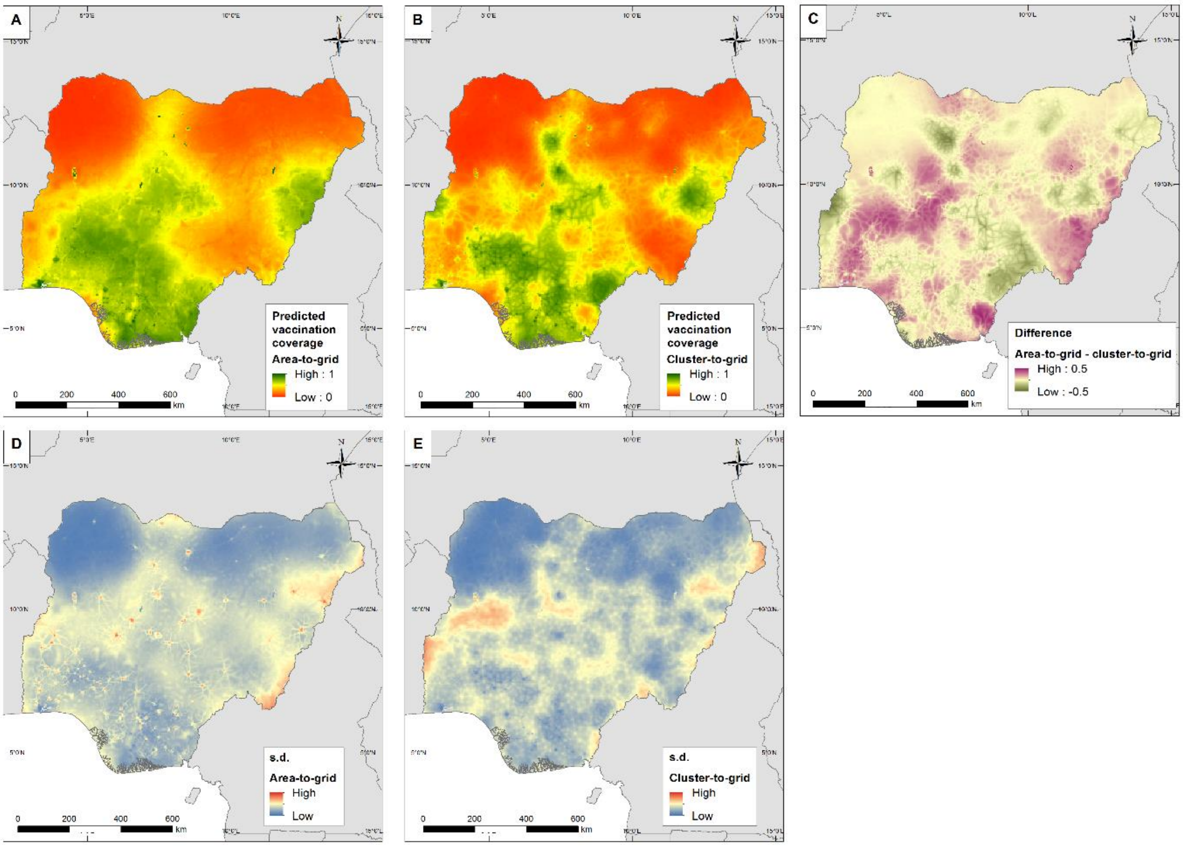

Figure S10: Predicted measles vaccination coverage and associated standard deviations at 5×5 km through using area-to-grid and cluster-to-grid approaches for Nigeria in 2014. The differences between the two approaches are shown in panel (C).

Table S4: Summary statistics of differences between area-to-grid and point-to-grid approaches

Country	Min.	1st Quartile	Mean	3rd Quartile	Max.
Cambodia	-0.230	-0.004	0.039	0.076	0.572
Mozambique	-0.358	0.012	0.052	0.100	0.345
Nigeria	-0.440	-0.014	0.051	0.118	0.513

Table S5: National estimates of numbers of children under 5 unvaccinated through using DHS administrative area coverage estimates in children aged 12-23 months (used as a proxy for coverage levels) and the 5 x 5 km area to grid and cluster to grid model predictions.

Country	DHS admin areas	Area to grid (95% CI)	Cluster to grid (95% CI)
Cambodia	362368	317815 (108644, 680786)	331906 (178542, 534179)
Mozambique	771020	897363 (200189, 2277703)	1020055 (367880, 1961305)
Nigeria	16025329	14844720 (9275806, 20696444)	15542358 (10904157, 19935291)

Mesh construction

The stochastic partial differential equation approach involves a triangulation of the spatial domain in order to approximate the latent Gaussian field. We constructed the mesh for this approximation in each case using the prediction grid points and a nonconvex hull bounding these points. In the simulation study, triangle maximum edge length was set to be 0.05 in the inner mesh (which is much smaller than the minimum spatial range of 0.3 used in data generation) and 0.2 in the outer mesh. The selection of these edge lengths was guided by the need to maintain a balance between the accuracy of the approximation and computational costs. For the applications in Sections 5 and 6, the same criterion was used. In Afghanistan, for example, the maximum triangle edge length was 0.35 degrees (which is approximately 2% of the maximum distance of ≈17 degrees between the prediction grids) in the inner mesh and 0.5 degrees in the outer mesh. The maximum edge lengths of the triangulations for other countries were chosen similarly. Figure S11 shows examples of the meshes used in this work.

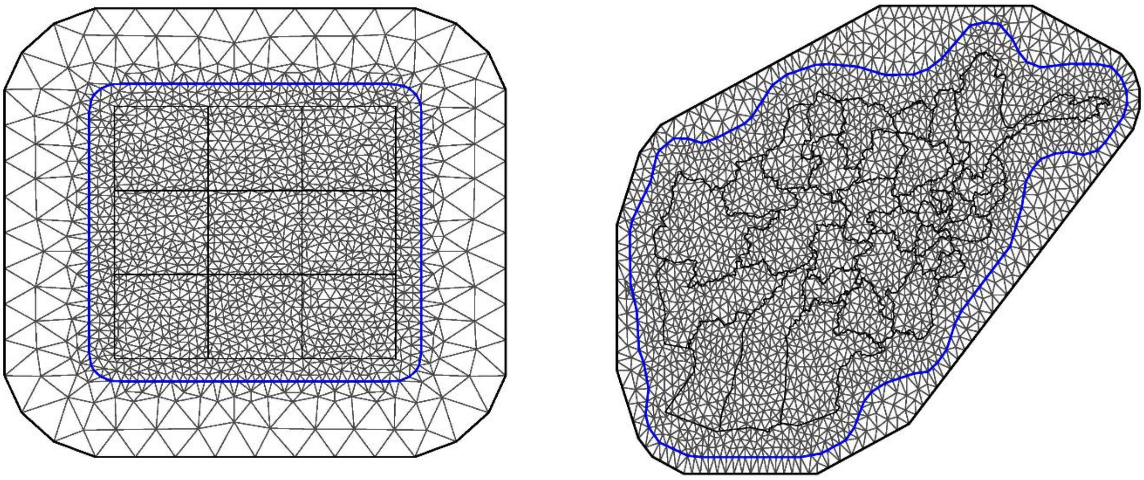

(a) (b)

Figure S11: Examples of the triangulations used in (a) the simulation study with $n_{\mathcal{A}} = 9$ and (b) vaccination coverage mapping for Afghanistan (b).

R code for analysis

```
######################################################################
## R code for "A spatial regression model for the disaggregation  ##
## of areal unit based data to high resolution grids with         ##
## application to vaccination coverage mapping"                   ##
## Authors: Utazi CE, Thorley J, Alegana VA, Ferrari MJ, Nilsen K,##
## Takahashi S, Metcalf CJE, Lessler J and Tatem AJ              ##
######################################################################

## Load required libraries
library(INLA); library(raster); library(maptools); library(gtools)
library(sp); library(spdep); library(parallel)

#### Prediction grid points
# cov.1: 1st covariate layer for prediction
# cov.2: 2nd covariate layer for prediction (Two covariates used for
#                                             illustration)
# coord.pred: matrix with coordinates of grid points for prediction
# ypred:  vector of binomial successes (NA) for the grid points
# npred:  vector of binomial samples (NA) for the grid points
# xpred1: vector of covariate values of the grid points
# xpred2: vector of covariate values of the grid points

#### Areal data
# area.poly.1: spatialPolygonsDataFrame object containing the areas
# area.poly:   spatialPolygons object containing the areas (from
#              area.poly.1)
# yarea:   vector of binomial successes for the areas
# narea:   vector of binomial samples for the areas
# xarea1:  vector of covariate values for the areas
# xarea2: vector of covariate values for the areas (these data must
          align with the shapefile)

# Polygon indexes/numbers for the grid points
grid.poly.no <- rep(NA, nrow(coord.pred))
for(i in 1:length(area.poly)){
 grid.poly.no[as.vector(which(!is.na(over(SpatialPoints(coord.pred),
 area.poly[i]))))] <- i
}

# Mesh (adjust arguments as necessary)
bnd  <- inla.nonconvex.hull(coord.pred,convex=-0.06)
mesh <- inla.mesh.2d(boundary = bnd, offset=c(0.1, 0.4),
        max.edge=c(0.35,0.5))

# Matern SPDE model object
nu     <- 1 #Matern smoothness parameter
ran.pr <- as.numeric(summary(dist(coopred))[3]) #Median of distances
                                           #between prediction grids
kap.pr <- sqrt(8*nu)/ran.pr
spde   <- inla.spde2.matern(mesh=meshfit, alpha=2,
                          B.tau=matrix(c(0, 1, 0),nrow=1,ncol=3),
                          B.kappa=matrix(c(0, 0, 1),nrow=1,ncol=3),
                          theta.prior.mean=c(0, log(kap.pr)),
                          theta.prior.prec=c(1, 1)) #Change priors
```

```
                                                      #as required

# Adjacency matrix for the areas
area.poly.nb  <- poly2nb(area.poly, snap = 1)
area.poly.adj <- nb2mat(area.poly.nb, style = "B")
area.poly.adj <- as(area.poly.adj, "dgTMatrix")

### Areal observations
# Extract covariate values for the areas from the covariate layers
xarea1 <- extract(cov.1, area.poly, fun=mean, na.rm=TRUE)
xarea2 <- extract(cov.2, area.poly, fun=mean, na.rm=TRUE)

# Mesh coordinates within the polygons
mesh.coord.in <-
mesh$loc[as.vector(which(!is.na(over(SpatialPoints(SpatialPoints(mes
h$loc)), area.poly)))),]

# Polygon indexes for the mesh coordinates
mesh.coord.poly.no <- numeric(nrow(mesh.coord.in))
for(i in 1:length(area.poly)){
      mesh.coord.poly.no[as.vector(which(!is.na(over(SpatialPoints
      (mesh.coord.in), area.poly[i]))))] <- i
}

Aarea <- inla.spde.make.A(mesh=mesh, loc=mesh.coord.in,
                      block=mesh.coord.poly.no, block.rescale="sum")
stack.area <- inla.stack(tag='areal',
                    data=list(y=yarea,n=narea),
                    A=list(Aarea,1,1),
             effects=list(s=1:spde$n.spde, sa=1:nrow(area.poly.1),
             data.frame(intercept=1, x1=xarea1, x2=xarea2)))

### Prediction grid points
Apred <- inla.spde.make.A(mesh=mesh, loc=coord.pred)
stack.pred <- inla.stack(tag='pred',
                       data=list(y=ypred,n=npred),
                       A=list(Apred,1,1),
                       effects=list(s=1:spde$n.spde,
                       sa=grid.poly.no, data.frame(intercept=1,
                       x1=xpred1, x2=xpred2)))

# Stack
stack.all <- inla.stack(stack.area, stack.pred)

# Fit model
# Note the prior on the precision for the areal random effect
formula  <- y ~ -1 + intercept + x1 + x2 + f(s, model=spde)
            + f(sa, model = "besagproper2", graph = area.poly.adj,
                hyper=list(prec=list(prior="loggamma",
                param=c(1,0.01))))

modfit <- inla(formula, data=inla.stack.data(stack.all),
               family="binomial", Ntrials = stack.all$data$data$n,
               control.predictor=list(compute=TRUE,
               A=inla.stack.A(stack.all), link=1),
               control.compute=list(dic=TRUE))
```

References

1. Utazi CE, Thorley J, Alegana VA, et al. High resolution age-structured mapping of childhood vaccination coverage in low and middle income countries. *Vaccine*. 2018; 36: 1583-91.
